# Supplementary figures and images for: Phytochemicals-linked food safety and human health protective benefits of the selected food-based botanicals
Source: PLoS One. 2024 Jul 29;19(7):e0307807. doi: 10.1371/journal.pone.0307807 (PMC11285910; doi:10.1371/journal.pone.0307807)

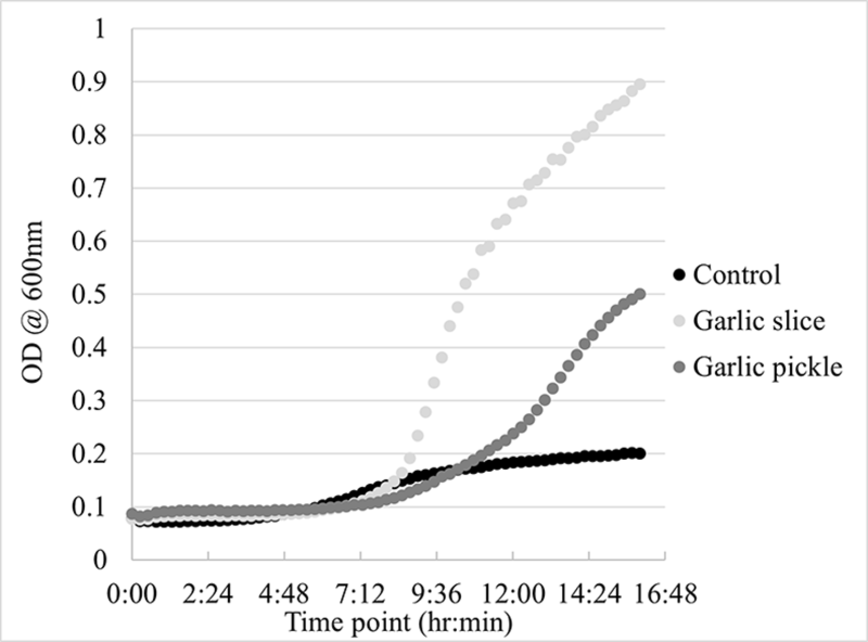

Supplement: S1 Fig — (TIF) [file pone.0307807.s001.tif]

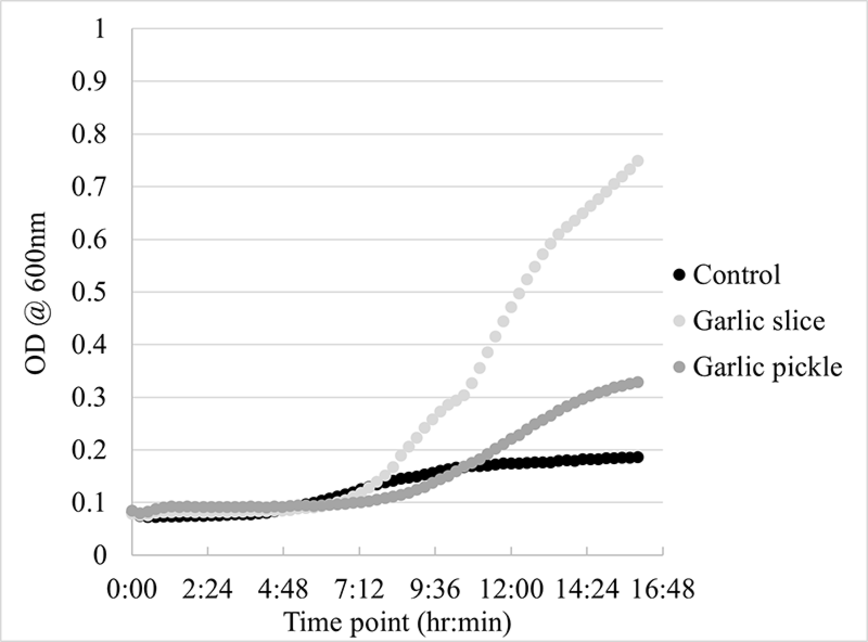

Supplement: S2 Fig — (TIF) [file pone.0307807.s002.tif]

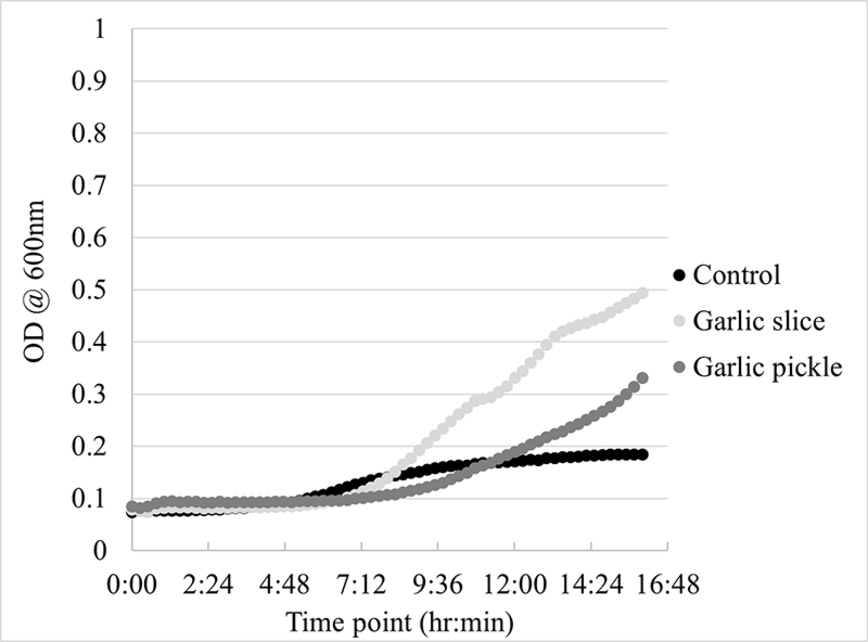

Supplement: S3 Fig — (TIF) [file pone.0307807.s003.tif]

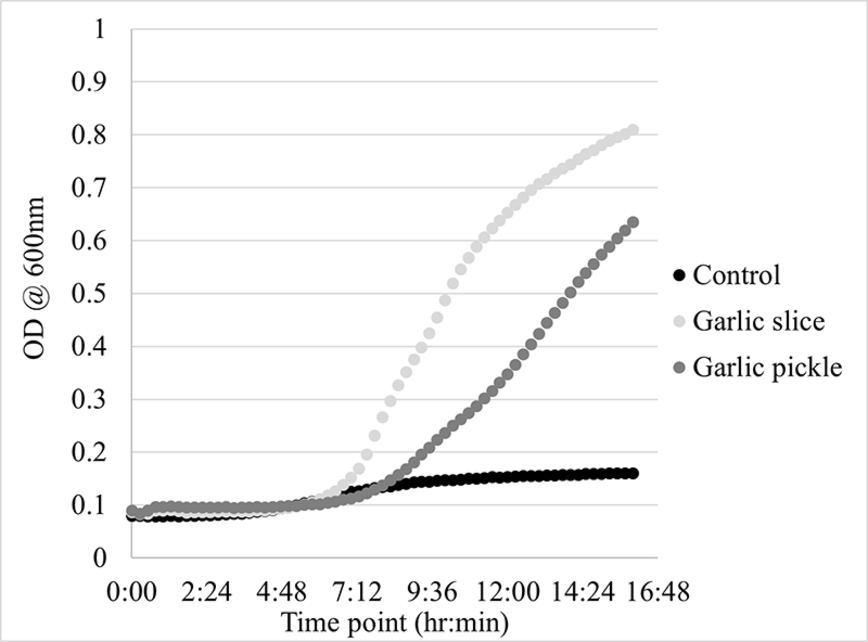

Supplement: S4 Fig — (TIF) [file pone.0307807.s004.tif]
