# Supplementary material for: Phytochemicals-linked food safety and human health protective benefits of the selected food-based botanicals
Source: PLoS One. 2024 Jul 29;19(7):e0307807. doi: 10.1371/journal.pone.0307807 (PMC11285910; doi:10.1371/journal.pone.0307807)
Supplement: S1 Table — (DOCX) [file pone.0307807.s005.docx]

S1 Table. Optical density values (OD 600mn) of *L. monocytogenes* (10403S) 1/2a in garlic slice and pickle extracts.

| **Time point (hr:min)** | **Control** | **Garlic slice** | **Garlic pickle** |
| --- | --- | --- | --- |
| 0:00 | 0.079 | 0.078 | 0.087 |
| 0:15 | 0.074 | 0.077 | 0.082 |
| 0:30 | 0.074 | 0.078 | 0.084 |
| 0:45 | 0.073 | 0.081 | 0.089 |
| 1:00 | 0.073 | 0.082 | 0.091 |
| 1:15 | 0.073 | 0.083 | 0.091 |
| 1:30 | 0.073 | 0.083 | 0.093 |
| 1:45 | 0.074 | 0.084 | 0.093 |
| 2:00 | 0.074 | 0.083 | 0.093 |
| 2:15 | 0.075 | 0.084 | 0.092 |
| 2:30 | 0.075 | 0.084 | 0.094 |
| 2:45 | 0.075 | 0.083 | 0.093 |
| 3:00 | 0.076 | 0.083 | 0.091 |
| 3:15 | 0.077 | 0.084 | 0.093 |
| 3:30 | 0.078 | 0.083 | 0.092 |
| 3:45 | 0.079 | 0.083 | 0.093 |
| 4:00 | 0.080 | 0.085 | 0.093 |
| 4:15 | 0.082 | 0.084 | 0.092 |
| 4:30 | 0.083 | 0.085 | 0.094 |
| 4:45 | 0.086 | 0.086 | 0.094 |
| 5:00 | 0.088 | 0.087 | 0.094 |
| 5:15 | 0.091 | 0.088 | 0.095 |
| 5:30 | 0.094 | 0.089 | 0.095 |
| 5:45 | 0.098 | 0.091 | 0.096 |
| 6:00 | 0.103 | 0.094 | 0.097 |
| 6:15 | 0.107 | 0.096 | 0.099 |
| 6:30 | 0.111 | 0.098 | 0.100 |
| 6:45 | 0.115 | 0.102 | 0.101 |
| 7:00 | 0.121 | 0.107 | 0.104 |
| 7:15 | 0.127 | 0.112 | 0.104 |
| 7:30 | 0.133 | 0.119 | 0.107 |
| 7:45 | 0.138 | 0.127 | 0.109 |
| 8:00 | 0.141 | 0.137 | 0.114 |
| 8:15 | 0.145 | 0.149 | 0.117 |
| 8:30 | 0.150 | 0.164 | 0.122 |
| 8:45 | 0.154 | 0.192 | 0.128 |
| 9:00 | 0.158 | 0.234 | 0.134 |
| 9:15 | 0.160 | 0.279 | 0.140 |
| 9:30 | 0.163 | 0.334 | 0.148 |
| **Time point (hr:min)** | **Control** | **Garlic slice** | **Garlic pickle** |
| 9:45 | 0.165 | 0.382 | 0.157 |
| 10:00 | 0.168 | 0.441 | 0.162 |
| 10:15 | 0.170 | 0.476 | 0.171 |
| 10:30 | 0.172 | 0.521 | 0.179 |
| 10:45 | 0.173 | 0.538 | 0.188 |
| 11:00 | 0.175 | 0.584 | 0.197 |
| 11:15 | 0.178 | 0.591 | 0.207 |
| 11:30 | 0.181 | 0.633 | 0.217 |
| 11:45 | 0.182 | 0.641 | 0.226 |
| 12:00 | 0.184 | 0.672 | 0.238 |
| 12:15 | 0.185 | 0.676 | 0.250 |
| 12:30 | 0.186 | 0.707 | 0.265 |
| 12:45 | 0.187 | 0.715 | 0.283 |
| 13:00 | 0.188 | 0.729 | 0.302 |
| 13:15 | 0.190 | 0.755 | 0.323 |
| 13:30 | 0.192 | 0.754 | 0.344 |
| 13:45 | 0.192 | 0.776 | 0.366 |
| 14:00 | 0.193 | 0.797 | 0.386 |
| 14:15 | 0.196 | 0.801 | 0.407 |
| 14:30 | 0.196 | 0.816 | 0.424 |
| 14:45 | 0.196 | 0.837 | 0.442 |
| 15:00 | 0.197 | 0.848 | 0.457 |
| 15:15 | 0.198 | 0.856 | 0.470 |
| 15:30 | 0.201 | 0.864 | 0.482 |
| 15:45 | 0.202 | 0.883 | 0.491 |
| 16:00 | 0.201 | 0.896 | 0.501 |
