# Supplementary material for: Phytochemicals-linked food safety and human health protective benefits of the selected food-based botanicals
Source: PLoS One. 2024 Jul 29;19(7):e0307807. doi: 10.1371/journal.pone.0307807 (PMC11285910; doi:10.1371/journal.pone.0307807)
Supplement: S2 Table — (DOCX) [file pone.0307807.s006.docx]

S2 Table. Optical density values (OD 600mn) of *L. monocytogenes* (FSL J1-0194) 1/2b in garlic slice and pickle extracts.

| **Time point (hr:min)** | **Control** | **Garlic slice** | **Garlic pickle** |
| --- | --- | --- | --- |
| 0:00 | 0.081 | 0.079 | 0.085 |
| 0:15 | 0.075 | 0.076 | 0.080 |
| 0:30 | 0.073 | 0.076 | 0.083 |
| 0:45 | 0.073 | 0.081 | 0.088 |
| 1:00 | 0.074 | 0.082 | 0.091 |
| 1:15 | 0.074 | 0.082 | 0.093 |
| 1:30 | 0.074 | 0.083 | 0.092 |
| 1:45 | 0.075 | 0.083 | 0.093 |
| 2:00 | 0.075 | 0.082 | 0.092 |
| 2:15 | 0.075 | 0.082 | 0.092 |
| 2:30 | 0.076 | 0.082 | 0.092 |
| 2:45 | 0.076 | 0.083 | 0.092 |
| 3:00 | 0.077 | 0.082 | 0.092 |
| 3:15 | 0.078 | 0.083 | 0.092 |
| 3:30 | 0.078 | 0.083 | 0.092 |
| 3:45 | 0.078 | 0.084 | 0.093 |
| 4:00 | 0.080 | 0.084 | 0.092 |
| 4:15 | 0.081 | 0.084 | 0.091 |
| 4:30 | 0.084 | 0.085 | 0.093 |
| 4:45 | 0.086 | 0.085 | 0.092 |
| 5:00 | 0.089 | 0.086 | 0.094 |
| 5:15 | 0.092 | 0.089 | 0.095 |
| 5:30 | 0.097 | 0.090 | 0.095 |
| 5:45 | 0.100 | 0.091 | 0.094 |
| 6:00 | 0.104 | 0.093 | 0.095 |
| 6:15 | 0.108 | 0.097 | 0.096 |
| 6:30 | 0.112 | 0.101 | 0.097 |
| 6:45 | 0.116 | 0.105 | 0.098 |
| 7:00 | 0.120 | 0.112 | 0.100 |
| 7:15 | 0.126 | 0.119 | 0.101 |
| 7:30 | 0.130 | 0.129 | 0.103 |
| 7:45 | 0.135 | 0.140 | 0.106 |
| 8:00 | 0.139 | 0.152 | 0.109 |
| 8:15 | 0.142 | 0.168 | 0.112 |
| 8:30 | 0.146 | 0.190 | 0.115 |
| 8:45 | 0.148 | 0.207 | 0.119 |
| 9:00 | 0.150 | 0.224 | 0.125 |
| 9:15 | 0.154 | 0.242 | 0.130 |
| 9:30 | 0.157 | 0.258 | 0.138 |
| **Time point (hr:min)** | **Control** | **Garlic slice** | **Garlic pickle** |
| 9:45 | 0.161 | 0.273 | 0.145 |
| 10:00 | 0.164 | 0.286 | 0.151 |
| 10:15 | 0.167 | 0.294 | 0.160 |
| 10:30 | 0.168 | 0.304 | 0.169 |
| 10:45 | 0.170 | 0.327 | 0.176 |
| 11:00 | 0.170 | 0.356 | 0.183 |
| 11:15 | 0.171 | 0.386 | 0.193 |
| 11:30 | 0.173 | 0.416 | 0.203 |
| 11:45 | 0.175 | 0.445 | 0.212 |
| 12:00 | 0.175 | 0.472 | 0.222 |
| 12:15 | 0.175 | 0.497 | 0.229 |
| 12:30 | 0.176 | 0.524 | 0.239 |
| 12:45 | 0.177 | 0.548 | 0.249 |
| 13:00 | 0.177 | 0.572 | 0.257 |
| 13:15 | 0.177 | 0.592 | 0.265 |
| 13:30 | 0.180 | 0.610 | 0.275 |
| 13:45 | 0.181 | 0.624 | 0.283 |
| 14:00 | 0.180 | 0.636 | 0.290 |
| 14:15 | 0.183 | 0.650 | 0.297 |
| 14:30 | 0.183 | 0.664 | 0.303 |
| 14:45 | 0.183 | 0.677 | 0.309 |
| 15:00 | 0.185 | 0.691 | 0.313 |
| 15:15 | 0.185 | 0.706 | 0.319 |
| 15:30 | 0.186 | 0.720 | 0.322 |
| 15:45 | 0.186 | 0.734 | 0.326 |
| 16:00 | 0.187 | 0.749 | 0.329 |
