# Supplementary material for: Phytochemicals-linked food safety and human health protective benefits of the selected food-based botanicals
Source: PLoS One. 2024 Jul 29;19(7):e0307807. doi: 10.1371/journal.pone.0307807 (PMC11285910; doi:10.1371/journal.pone.0307807)
Supplement: S3 Table — (DOCX) [file pone.0307807.s007.docx]

S3 Table. Optical density values (OD 600mn) of *L. monocytogenes* 1/2a (FSL F2-0515) in garlic slice and pickle extracts.

| **Time point (hr:min)** | **Control** | **Garlic slice** | **Garlic pickle** |
| --- | --- | --- | --- |
| 0:00 | 0.075 | 0.079 | 0.086 |
| 0:15 | 0.077 | 0.077 | 0.082 |
| 0:30 | 0.076 | 0.076 | 0.085 |
| 0:45 | 0.077 | 0.081 | 0.091 |
| 1:00 | 0.077 | 0.082 | 0.094 |
| 1:15 | 0.077 | 0.083 | 0.095 |
| 1:30 | 0.077 | 0.083 | 0.093 |
| 1:45 | 0.077 | 0.083 | 0.094 |
| 2:00 | 0.078 | 0.082 | 0.094 |
| 2:15 | 0.078 | 0.081 | 0.092 |
| 2:30 | 0.079 | 0.082 | 0.092 |
| 2:45 | 0.079 | 0.082 | 0.094 |
| 3:00 | 0.081 | 0.082 | 0.092 |
| 3:15 | 0.082 | 0.083 | 0.093 |
| 3:30 | 0.082 | 0.083 | 0.093 |
| 3:45 | 0.084 | 0.084 | 0.093 |
| 4:00 | 0.085 | 0.083 | 0.093 |
| 4:15 | 0.086 | 0.084 | 0.093 |
| 4:30 | 0.088 | 0.084 | 0.094 |
| 4:45 | 0.090 | 0.085 | 0.094 |
| 5:00 | 0.093 | 0.086 | 0.093 |
| 5:15 | 0.096 | 0.087 | 0.095 |
| 5:30 | 0.100 | 0.088 | 0.095 |
| 5:45 | 0.104 | 0.090 | 0.095 |
| 6:00 | 0.107 | 0.092 | 0.096 |
| 6:15 | 0.112 | 0.095 | 0.097 |
| 6:30 | 0.117 | 0.097 | 0.097 |
| 6:45 | 0.122 | 0.102 | 0.098 |
| 7:00 | 0.127 | 0.108 | 0.100 |
| 7:15 | 0.131 | 0.114 | 0.101 |
| 7:30 | 0.135 | 0.121 | 0.103 |
| 7:45 | 0.138 | 0.130 | 0.105 |
| 8:00 | 0.141 | 0.139 | 0.107 |
| 8:15 | 0.144 | 0.151 | 0.109 |
| 8:30 | 0.146 | 0.165 | 0.112 |
| 8:45 | 0.149 | 0.178 | 0.115 |
| 9:00 | 0.152 | 0.193 | 0.118 |
| 9:15 | 0.155 | 0.207 | 0.122 |
| 9:30 | 0.158 | 0.221 | 0.126 |
| **Time point (hr:min)** | **Control** | **Garlic slice** | **Garlic pickle** |
| 9:45 | 0.16 | 0.234 | 0.131 |
| 10:00 | 0.162 | 0.248 | 0.137 |
| 10:15 | 0.164 | 0.262 | 0.143 |
| 10:30 | 0.164 | 0.275 | 0.149 |
| 10:45 | 0.166 | 0.287 | 0.158 |
| 11:00 | 0.168 | 0.291 | 0.164 |
| 11:15 | 0.168 | 0.294 | 0.169 |
| 11:30 | 0.17 | 0.304 | 0.177 |
| 11:45 | 0.171 | 0.315 | 0.184 |
| 12:00 | 0.172 | 0.331 | 0.190 |
| 12:15 | 0.173 | 0.344 | 0.196 |
| 12:30 | 0.176 | 0.361 | 0.204 |
| 12:45 | 0.175 | 0.377 | 0.210 |
| 13:00 | 0.178 | 0.395 | 0.218 |
| 13:15 | 0.178 | 0.411 | 0.224 |
| 13:30 | 0.180 | 0.421 | 0.230 |
| 13:45 | 0.180 | 0.427 | 0.237 |
| 14:00 | 0.181 | 0.433 | 0.243 |
| 14:15 | 0.183 | 0.436 | 0.251 |
| 14:30 | 0.183 | 0.443 | 0.259 |
| 14:45 | 0.184 | 0.448 | 0.267 |
| 15:00 | 0.185 | 0.457 | 0.276 |
| 15:15 | 0.186 | 0.466 | 0.287 |
| 15:30 | 0.185 | 0.475 | 0.300 |
| 15:45 | 0.185 | 0.484 | 0.314 |
| 16:00 | 0.185 | 0.495 | 0.331 |
