# Supplementary material for: Phytochemicals-linked food safety and human health protective benefits of the selected food-based botanicals
Source: PLoS One. 2024 Jul 29;19(7):e0307807. doi: 10.1371/journal.pone.0307807 (PMC11285910; doi:10.1371/journal.pone.0307807)
Supplement: S4 Table — (DOCX) [file pone.0307807.s008.docx]

S4 Table. Optical density values (OD 600mn) of *L. monocytogenes* 4b (H7858) in garlic slice and pickle extracts.

| **Time point (hr:min)** | **Control** | **Garlic slice** | **Garlic pickle** |
| --- | --- | --- | --- |
| 0:00 | 0.080 | 0.087 | 0.090 |
| 0:15 | 0.080 | 0.083 | 0.084 |
| 0:30 | 0.079 | 0.084 | 0.090 |
| 0:45 | 0.079 | 0.090 | 0.097 |
| 1:00 | 0.079 | 0.090 | 0.097 |
| 1:15 | 0.080 | 0.091 | 0.098 |
| 1:30 | 0.079 | 0.089 | 0.097 |
| 1:45 | 0.080 | 0.086 | 0.096 |
| 2:00 | 0.080 | 0.087 | 0.096 |
| 2:15 | 0.081 | 0.087 | 0.096 |
| 2:30 | 0.081 | 0.087 | 0.096 |
| 2:45 | 0.082 | 0.088 | 0.096 |
| 3:00 | 0.083 | 0.088 | 0.097 |
| 3:15 | 0.084 | 0.088 | 0.095 |
| 3:30 | 0.084 | 0.088 | 0.096 |
| 3:45 | 0.086 | 0.088 | 0.096 |
| 4:00 | 0.088 | 0.090 | 0.097 |
| 4:15 | 0.089 | 0.090 | 0.096 |
| 4:30 | 0.091 | 0.092 | 0.097 |
| 4:45 | 0.094 | 0.094 | 0.098 |
| 5:00 | 0.097 | 0.095 | 0.099 |
| 5:15 | 0.100 | 0.098 | 0.099 |
| 5:30 | 0.104 | 0.102 | 0.101 |
| 5:45 | 0.107 | 0.106 | 0.102 |
| 6:00 | 0.111 | 0.112 | 0.102 |
| 6:15 | 0.115 | 0.119 | 0.105 |
| 6:30 | 0.119 | 0.127 | 0.107 |
| 6:45 | 0.123 | 0.138 | 0.111 |
| 7:00 | 0.126 | 0.152 | 0.113 |
| 7:15 | 0.127 | 0.169 | 0.117 |
| 7:30 | 0.130 | 0.196 | 0.123 |
| 7:45 | 0.132 | 0.232 | 0.130 |
| 8:00 | 0.134 | 0.266 | 0.137 |
| 8:15 | 0.135 | 0.297 | 0.147 |
| 8:30 | 0.138 | 0.327 | 0.157 |
| 8:45 | 0.140 | 0.352 | 0.168 |
| 9:00 | 0.143 | 0.375 | 0.181 |
| 9:15 | 0.144 | 0.398 | 0.196 |
| 9:30 | 0.144 | 0.425 | 0.209 |
| **Time point (hr:min)** | **Control** | **Garlic slice** | **Garlic pickle** |
| 9:45 | 0.146 | 0.455 | 0.224 |
| 10:00 | 0.147 | 0.487 | 0.237 |
| 10:15 | 0.147 | 0.519 | 0.25 |
| 10:30 | 0.148 | 0.546 | 0.262 |
| 10:45 | 0.150 | 0.568 | 0.274 |
| 11:00 | 0.150 | 0.589 | 0.287 |
| 11:15 | 0.151 | 0.606 | 0.302 |
| 11:30 | 0.153 | 0.623 | 0.316 |
| 11:45 | 0.152 | 0.638 | 0.332 |
| 12:00 | 0.153 | 0.653 | 0.348 |
| 12:15 | 0.154 | 0.668 | 0.366 |
| 12:30 | 0.155 | 0.682 | 0.385 |
| 12:45 | 0.155 | 0.696 | 0.404 |
| 13:00 | 0.155 | 0.708 | 0.424 |
| 13:15 | 0.156 | 0.717 | 0.445 |
| 13:30 | 0.156 | 0.727 | 0.464 |
| 13:45 | 0.157 | 0.736 | 0.483 |
| 14:00 | 0.157 | 0.744 | 0.502 |
| 14:15 | 0.157 | 0.754 | 0.522 |
| 14:30 | 0.159 | 0.764 | 0.539 |
| 14:45 | 0.159 | 0.771 | 0.556 |
| 15:00 | 0.159 | 0.781 | 0.574 |
| 15:15 | 0.160 | 0.789 | 0.589 |
| 15:30 | 0.160 | 0.796 | 0.604 |
| 15:45 | 0.160 | 0.802 | 0.619 |
| 16:00 | 0.160 | 0.810 | 0.635 |
