# Supplementary material for: Phytochemicals-linked food safety and human health protective benefits of the selected food-based botanicals
Source: PLoS One. 2024 Jul 29;19(7):e0307807. doi: 10.1371/journal.pone.0307807 (PMC11285910; doi:10.1371/journal.pone.0307807)
Supplement: S5 Table — (DOCX) [file pone.0307807.s009.docx]

S5 Table. Values of total soluble phenolic content of the selected botanical extracts.

| **Sample^a^** | **Replicates** | | | | | | | | | | | | **Average** | **Standard error** |
| --- | --- | --- | --- | --- | --- | --- | --- | --- | --- | --- | --- | --- | --- | --- |
|  | **1** | **2** | **3** | **4** | **5** | **6** | **7** | **8** | **9** | **10** | **11** | **12** |  |  |
| Clove powder | 91.88 | 93.38 | 95.83 | 90.83 | 93.83 | 93.50 | 80.33 | 81.88 | 80.42 | 84.67 | 84.67 | 84.83 | 88.00 | 1.66 |
| Amla powder | 126.75 | 134.88 | 125.46 | 134.58 | 134.25 | 131.29 | 135.50 | 131.75 | 133.79 | 134.08 | 136.71 | 132.67 | 132.64 | 0.99 |
| Amla slices | 39.62 | 38.90 | 39.20 | 39.97 | 39.55 | 40.07 | 38.67 | 36.75 | 36.52 | 35.68 | 36.08 | 37.07 | 38.17 | 0.47 |
| Amla pickle | 13.79 | 13.75 | 13.92 | 13.46 | 13.54 | 14.04 | 13.17 | 12.58 | 12.54 | 13.17 | 13.17 | 12.88 | 13.33 | 0.14 |
| Garlic slices | 0.70 | 0.73 | 0.72 | 0.78 | 0.78 | 0.78 | 0.63 | 0.65 | 0.73 | 0.68 | 0.67 | 0.85 | 0.73 | 0.02 |
| Garlic pickle | 2.00 | 1.83 | 1.96 | 1.50 | 1.58 | 1.50 | 1.63 | 1.63 | 1.79 | 1.63 | 1.63 | 1.67 | 1.69 | 0.05 |
| Kokum powder | 14.33 | 14.29 | 14.13 | 13.58 | 13.92 | 13.67 | 11.04 | 10.92 | 11.08 | 13.75 | 13.67 | 13.79 | 13.18 | 0.38 |
| Kokum slices | 3.93 | 4.10 | 4.12 | 4.02 | 4.20 | 4.32 | 3.83 | 3.95 | 4.18 | 3.67 | 3.68 | 3.83 | 3.99 | 0.06 |

^a^ Values expressed in milligram gallic acid equivalents per gram fresh or dry weight (mg GAE/g FW or DW).
