# Supplementary material for: Phytochemicals-linked food safety and human health protective benefits of the selected food-based botanicals
Source: PLoS One. 2024 Jul 29;19(7):e0307807. doi: 10.1371/journal.pone.0307807 (PMC11285910; doi:10.1371/journal.pone.0307807)
Supplement: S6 Table — (DOCX) [file pone.0307807.s010.docx]

S6 Table. Values of ABTS- based antioxidant activity of the selected botanical extracts.

| **Sample^a^** | **Replicates** | | | | | | | | | | | | **Average** | **Standard error** |
| --- | --- | --- | --- | --- | --- | --- | --- | --- | --- | --- | --- | --- | --- | --- |
|  | **1** | **2** | **3** | **4** | **5** | **6** | **7** | **8** | **9** | **10** | **11** | **12** |  |  |
| Clove powder | 0.52 | 0.52 | 0.52 | 0.52 | 0.52 | 0.52 | 0.48 | 0.49 | 0.50 | 0.50 | 0.49 | 0.49 | 0.50 | 0.00 |
| Amla powder | 0.52 | 0.52 | 0.52 | 0.52 | 0.52 | 0.52 | 0.48 | 0.51 | 0.51 | 0.46 | 0.50 | 0.50 | 0.51 | 0.01 |
| Amla slices | 0.52 | 0.52 | 0.52 | 0.52 | 0.52 | 0.52 | 0.51 | 0.52 | 0.52 | 0.52 | 0.52 | 0.51 | 0.52 | 0.00 |
| Amla pickle | 0.52 | 0.52 | 0.52 | 0.52 | 0.52 | 0.52 | 0.52 | 0.52 | 0.51 | 0.52 | 0.52 | 0.52 | 0.52 | 0.00 |
| Garlic slices | 0.51 | 0.52 | 0.52 | 0.49 | 0.50 | 0.51 | 0.46 | 0.48 | 0.48 | 0.48 | 0.48 | 0.49 | 0.49 | 0.01 |
| Garlic pickle | 0.51 | 0.51 | 0.51 | 0.50 | 0.50 | 0.51 | 0.49 | 0.50 | 0.50 | 0.49 | 0.50 | 0.50 | 0.50 | 0.00 |
| Kokum powder | 0.49 | 0.48 | 0.49 | 0.49 | 0.48 | 0.50 | 0.50 | 0.51 | 0.50 | 0.51 | 0.50 | 0.51 | 0.50 | 0.00 |
| Kokum slices | 0.49 | 0.49 | 0.52 | 0.49 | 0.50 | 0.51 | 0.51 | 0.51 | 0.52 | 0.51 | 0.51 | 0.51 | 0.51 | 0.00 |

^a^ 2, 2-Azino-bis-(3-ethylbenzthiazoline-6-sulfonic acid) (ABTS) scavenging activity expressed in millimolar Trolox equivalents (mm TE).
