# Supplementary material for: Phytochemicals-linked food safety and human health protective benefits of the selected food-based botanicals
Source: PLoS One. 2024 Jul 29;19(7):e0307807. doi: 10.1371/journal.pone.0307807 (PMC11285910; doi:10.1371/journal.pone.0307807)
Supplement: S7 Table — (DOCX) [file pone.0307807.s011.docx]

S7 Table. Values of DPPH- based antioxidant activity of the selected botanical extracts.

| **Sample^a^** | **Replicates** | | | | | | | | | | | | **Average** | **Standard error** |
| --- | --- | --- | --- | --- | --- | --- | --- | --- | --- | --- | --- | --- | --- | --- |
|  | **1** | **2** | **3** | **4** | **5** | **6** | **7** | **8** | **9** | **10** | **11** | **12** |  |  |
| Clove powder | 0.06 | 0.05 | 0.03 | 0.13 | 0.13 | 0.07 | 0.12 | 0.09 | 0.13 | 0.12 | 0.15 | 0.12 | 0.10 | 0.01 |
| Amla powder | 0.36 | 0.35 | 0.35 | 0.35 | 0.35 | 0.35 | 0.35 | 0.35 | 0.35 | 0.36 | 0.36 | 0.36 | 0.35 | 0.00 |
| Amla slices | 0.39 | 0.39 | 0.39 | 0.39 | 0.39 | 0.39 | 0.40 | 0.40 | 0.40 | 0.40 | 0.40 | 0.40 | 0.39 | 0.00 |
| Amla pickle | 0.38 | 0.38 | 0.38 | 0.38 | 0.38 | 0.38 | 0.38 | 0.38 | 0.38 | 0.38 | 0.38 | 0.39 | 0.38 | 0.00 |
| Garlic slices | 0.10 | 0.11 | 0.10 | 0.12 | 0.12 | 0.12 | 0.07 | 0.07 | 0.07 | 0.08 | 0.08 | 0.09 | 0.09 | 0.01 |
| Garlic pickle | 0.26 | 0.25 | 0.26 | 0.26 | 0.27 | 0.28 | 0.23 | 0.23 | 0.23 | 0.21 | 0.22 | 0.24 | 0.24 | 0.01 |
| Kokum powder | 0.37 | 0.37 | 0.37 | 0.37 | 0.37 | 0.37 | 0.37 | 0.37 | 0.37 | 0.37 | 0.37 | 0.38 | 0.37 | 0.00 |
| Kokum slices | 0.36 | 0.36 | 0.36 | 0.36 | 0.36 | 0.36 | 0.36 | 0.36 | 0.36 | 0.35 | 0.35 | 0.36 | 036 | 0.00 |

^a^ 2, 2-Dipheny-1-Picryl Hydrazyl (DPPH) scavenging activity expressed in millimolar Trolox equivalents (mm TE).
