# Supplementary material for: Phytochemicals-linked food safety and human health protective benefits of the selected food-based botanicals
Source: PLoS One. 2024 Jul 29;19(7):e0307807. doi: 10.1371/journal.pone.0307807 (PMC11285910; doi:10.1371/journal.pone.0307807)
Supplement: S8 Table — (DOCX) [file pone.0307807.s012.docx]

S8 Table. Values of angiotensin-I-converting enzyme (ACE) inhibitory activity of the selected botanical extracts expressed as percentages (%)

| **Botanical extracts**^a^ | **Undiluted** | **Half-diluted** | **One-fifth diluted** |
| --- | --- | --- | --- |
| Clove powder | 91.4 ± 1.43 | 84.4 ± 1.60 | 57.0 ± 1.9 |
| Amla powder | 100 ± 0.0 | 100 ± 0.0 | 100 ± 0.0 |
| Amla slice | 98.0 ± 0.8 | 88.5 ± 4.4 | 2.3 ± 0.9 |
| Amla pickle | 93.1 ± 0.2 | 45.9 ± 4.7 | 0.0 ± 0.0 |
| Garlic slice | 90.1 ± 2.1 | 89.3 ± 2.1 | 86.1 ± 1.9 |
| Garlic pickle | 93.3 ± 0.9 | 57.5 ± 2.9 | 6.6 ± 2.1 |
| Kokum powder | 100 ± 0.0 | 100 ± 0.0 | 98.7 ± 0.0 |
| Kokum slice | 100 ± 0.0 | 100 ± 0.0 | 98.6 ± 0.1 |

^a^ Mean ± standard error
