# Supplementary material for: Phytochemicals-linked food safety and human health protective benefits of the selected food-based botanicals
Source: PLoS One. 2024 Jul 29;19(7):e0307807. doi: 10.1371/journal.pone.0307807 (PMC11285910; doi:10.1371/journal.pone.0307807)

PLOS ONE

Phytochemicals-Linked Dual Functional Food Safety and Human Health Protective Benefits of Select Food-Based Botanicals

--Manuscript Draft--

**Manuscript Number:**	PONE-D-24-12227
**Article Type:**	Research Article
**Full Title:**	Phytochemicals-Linked Dual Functional Food Safety and Human Health Protective Benefits of Select Food-Based Botanicals
**Short Title:**	Dual Functional Botanicals for Food Safety and Dietary Chronic Disease Solutions
**Corresponding Author:**	Kalidas Shetty, Ph.D<br>North Dakota State University<br>Fargo, North Dakota UNITED STATES
**Keywords:**	Antimicrobials; Glycemic Control; Dual Functional Phytochemicals; Non-Communicable Chromic Disease; Food Safety
**Abstract:**	Phytochemicals-rich food-based botanicals including traditional or under-utilized plant-based ingredients can serve a dual functional role to help counter microbial spoilage or food contamination with bacterial pathogens, while also addressing the rise of diet-linked non-communicable chronic diseases (NCDs) such as type 2 diabetes and the associated chronic hypertension and oxidative stress. Hence the screening of these food-based botanicals for their phenolic content and profile, as well as antimicrobial, antioxidant, anti-hyperglycemic and anti-hypertensive properties has relevant merit. Using in vitro assay models, hot water extracts of different forms (slice, pickle, or powder) of amla (Phyllanthus emblica), clove (Syzygium aromaticum), kokum (Garcinia indica), and garlic (Allium sativum) were analyzed for their total soluble phenolic content and phenolic profile as well as antimicrobial activity against strains of Salmonella Enteritidis, Listeria monocytogenes, and Escherichia coli that are associated with food-borne disease outbreaks. In addition, the antioxidant, anti-hyperglycemic and anti-hypertensive activity of the extracts was also determined using in vitro assay models, with the goal of establishing a dual functional role of the food safety and health protective benefits of these botanicals. A high baseline TSP content was observed in all the extracts and the major phenolic phytochemicals detected were gallic, cinnamic, ellagic, benzoic, dihydroxybenzoic, protocatechuic, and p-coumaric acid along with catechin and rutin. All extracts displayed significant antimicrobial activity against most of the bacterial strains tested and the antimicrobial activity was specific for each strain targeted in this study. Furthermore, significant antioxidant, anti-hyperglycemic and antihypertensive activity were observed among the botanical extracts, especially among the amla and kokum extracts. These results indicate that phytochemicals enriched botanicals, including amla and kokum, can be integrated into modern-day food preservation and dietary support strategies aimed at improving the food safety and health protective benefits of the food matrix.
**Order of Authors:**	Ashish Christopher, Ph.D
	Kalidas Shetty, Ph.D
**Additional Information:**	
**Question**	**Response**
**Financial Disclosure**<br><br>Enter a financial disclosure statement that describes the sources of funding for the work included in this submission. Review the submission guidelines for detailed requirements. View published research articles from *PLOS ONE* for specific examples.	The author(s) received no specific funding for this work.

Powered by Editorial Manager® and ProduXion Manager® from Aries Systems Corporation

This statement is required for submission and **will appear in the published article** if the submission is accepted. Please make sure it is accurate.<br><br>**Funded studies**<br>Enter a statement with the following details:<br>• Initials of the authors who received each award<br>• Grant numbers awarded to each author<br>• The full name of each funder<br>• URL of each funder website<br>• Did the sponsors or funders play any role in the study design, data collection and analysis, decision to publish, or preparation of the manuscript?<br><br>Did you receive funding for this work?	
**Competing Interests**<br><br>Use the instructions below to enter a competing interest statement for this submission. On behalf of all authors, disclose any competing interests that could be perceived to bias this work—acknowledging all financial support and any other relevant financial or non-financial competing interests.<br><br>This statement is required for submission and **will appear in the published article** if the submission is accepted. Please make sure it is accurate and that any funding sources listed in your Funding Information later in the submission form are also declared in your Financial Disclosure statement.<br><br>View published research articles from *PLOS ONE* for specific examples.	The authors have declared that no competing interests exist.

Powered by Editorial Manager® and ProduXion Manager® from Aries Systems Corporation

**NO authors have competing interests**<br><br>Enter: *The authors have declared that no competing interests exist.*<br><br>**Authors with competing interests**<br><br>Enter competing interest details beginning with this statement:<br><br>*I have read the journal's policy and the authors of this manuscript have the following competing interests: [insert competing interests here]*<br><br>* typeset	
**Ethics Statement**<br><br>Enter an ethics statement for this submission. This statement is required if the study involved:<br><br>• Human participants<br>• Human specimens or tissue<br>• Vertebrate animals or cephalopods<br>• Vertebrate embryos or tissues<br>• Field research<br><br>Write "N/A" if the submission does not require an ethics statement.<br><br>General guidance is provided below. Consult the submission guidelines for detailed instructions. **Make sure that all information entered here is included in the Methods section of the manuscript.**	NA

**Format for specific study types**<br><br>**Human Subject Research (involving human participants and/or tissue)**<br>• Give the name of the institutional review board or ethics committee that approved the study<br>• Include the approval number and/or a statement indicating approval of this research<br>• Indicate the form of consent obtained (written/oral) or the reason that consent was not obtained (e.g. the data were analyzed anonymously)<br><br>**Animal Research (involving vertebrate animals, embryos or tissues)**<br>• Provide the name of the Institutional Animal Care and Use Committee (IACUC) or other relevant ethics board that reviewed the study protocol, and indicate whether they approved this research or granted a formal waiver of ethical approval<br>• Include an approval number if one was obtained<br>• If the study involved *non-human primates*, add *additional details* about animal welfare and steps taken to ameliorate suffering<br>• If anesthesia, euthanasia, or any kind of animal sacrifice is part of the study, include briefly which substances and/or methods were applied<br><br>**Field Research**<br><br>Include the following details if this study involves the collection of plant, animal, or other materials from a natural setting:<br>• Field permit number<br>• Name of the institution or relevant body that granted permission	
**Data Availability**<br><br>Authors are required to make all data underlying the findings described fully available, without restriction, and from the time of publication. PLOS allows rare exceptions to address legal and ethical concerns. See the PLOS Data Policy and FAQ for detailed information.	Yes - all data are fully available without restriction

Powered by Editorial Manager® and ProduXion Manager® from Aries Systems Corporation

A Data Availability Statement describing where the data can be found is required at submission. Your answers to this question constitute the Data Availability Statement and **will be published in the article**, if accepted.<br><br>**Important:** Stating 'data available on request from the author' is not sufficient. If your data are only available upon request, select 'No' for the first question and explain your exceptional situation in the text box.<br><br>Do the authors confirm that all data underlying the findings described in their manuscript are fully available without restriction?	
**Describe where the data may be found in full sentences. If you are copying our sample text, replace any instances of XXX with the appropriate details.**<br><br>• If the data are **held or will be held in a public repository**, include URLs, accession numbers or DOIs. If this information will only be available after acceptance, indicate this by ticking the box below. For example: *All XXX files are available from the XXX database (accession number(s) XXX, XXX.).*<br>• If the data are all contained **within the manuscript and/or Supporting Information files**, enter the following: *All relevant data are within the manuscript and its Supporting Information files.*<br>• If neither of these applies but you are able to provide **details of access elsewhere**, with or without limitations, please do so. For example:<br><br>*Data cannot be shared publicly because of [XXX]. Data are available from the XXX Institutional Data Access / Ethics Committee (contact via XXX) for researchers who meet the criteria for access to confidential data.*<br><br>*The data underlying the results presented in the study are available from (include the name of the third party*	All relevant data are within the manuscript and its Supporting Information files.

Powered by Editorial Manager® and ProduXion Manager® from Aries Systems Corporation

and contact information or URL).<br>• This text is appropriate if the data are owned by a third party and authors do not have permission to share the data.<br><br>* typeset	
Additional data availability information:	Tick here if your circumstances are not covered by the questions above and you need the journal's help to make your data available.

Powered by Editorial Manager® and ProduXion Manager® from Aries Systems Corporation

Manuscript Click here to access/download;Manuscript;Manuscript Text PLOS ONE.docx

Phytochemicals-Linked Dual Functional Food Safety and Human Health Protective Benefits of Select Food-Based Botanicals

Ashish Christopher and Kalidas Shetty[1*]

[1]Department of Plant Sciences, North Dakota State University, Fargo, North Dakota, United States of America

[*]Corresponding author

E-mail: kalidas.shetty@ndsu.edu

Short Title: Dual Functional Botanicals for Food Safety and Dietary Chronic Disease Solutions

Key Words: Antimicrobials, Glycemic Control, Dual Functional Phytochemicals, Non-Communicable Chromic Disease, Food Safety,

Abstract

Phytochemicals-rich food-based botanicals including traditional or under-utilized plant-based ingredients can serve a dual functional role to help counter microbial spoilage or food contamination with bacterial pathogens, while also addressing the rise of diet-linked non-communicable chronic diseases (NCDs) such as type 2 diabetes and the associated chronic hypertension and oxidative stress. Hence the screening of these food-based botanicals for their phenolic content and profile, as well as antimicrobial, antioxidant, anti-hyperglycemic and anti-hypertensive properties has relevant merit. Using *in vitro* assay models, hot water extracts of different forms (slice, pickle, or powder) of amla (*Phyllanthus emblica*), clove (*Syzygium aromaticum*), kokum (*Garcinia indica*), and garlic (*Allium sativum*) were analyzed for their total soluble phenolic content and phenolic profile as well as antimicrobial activity against strains of *Salmonella Enteritidis*, *Listeria monocytogenes*, and *Escherichia coli* that are associated with food-borne disease outbreaks. In addition, the antioxidant, anti-hyperglycemic and anti-hypertensive activity of the extracts was also determined using *in vitro* assay models, with the goal of establishing a dual functional role of the food safety and health protective benefits of these botanicals. A high baseline TSP content was observed in all the extracts and the major phenolic phytochemicals detected were gallic, cinnamic, ellagic, benzoic, dihydroxybenzoic, protocatechuic, and *p*-coumaric acid along with catechin and rutin. All extracts displayed significant antimicrobial activity against most of the bacterial strains tested and the antimicrobial activity was specific for each strain targeted in this study. Furthermore, significant antioxidant, anti-hyperglycemic and antihypertensive activity were observed among the botanical extracts, especially among the amla and kokum extracts. These results indicate that phytochemicals enriched botanicals, including amla and kokum, can be integrated into modern-day food

preservation and dietary support strategies aimed at improving the food safety and health protective benefits of the food matrix.

1. Introduction

The screening of phytochemicals-rich food-based botanicals including traditional or under-utilized plant ingredient sources is important to address foodborne diseases that commonly occur due to food contamination with bacterial pathogens. These same phytochemicals rich food botanicals also potentially have dual functional benefits in dietary support strategies that can be aimed at the prevention or management of diet-linked non-communicable chronic diseases (NCDs). Amla (*Emblica officinalis* Gaertn or *Phyllanthus emblica* Linn), commonly known as Indian gooseberry, is an important traditional fruit and medicinal plant in the Indian Subcontinent and other parts of the South-East Asia. This underutilized fruit is widely found in India, Pakistan, Sri Lanka, China, Uzbekistan, and Malaysia [1]. Amla is known to possess a wide range of bioactive properties including antioxidant, antidiabetic, and antimicrobial activities [2-8]. *In vitro* studies have shown amla to possess antimicrobial activity against gram-positive (*Staphylococcus*, *Micrococcus*, and *Bacillus*) and gram-negative (*E. coli* and *Salmonella*) bacterial pathogens [9-13]. Due to its high bioactivity, amla has the potential to be utilized as a dual functional food ingredient in different food formulations for the improvement of food safety and human health benefits. Previous research has targeted incorporation of amla into mixed fruit beverages, pan bread, and chicken feed to improve functional properties [14-18].

Another phytochemicals-rich botanical, kokum (*Garcinia indica*) or mangosteen is an indigenous fruit commonly found in India and has many phytopharmacological properties with

wider food, pharmaceutical, and industrial applications [19-23]. The major phytochemicals present in kokum that are responsible for their wider bioactivity include garcinol, hydroxycitric acid (HCA), cyanidin-3-sambubioside, and cyanidin-3-glucoside [20-22, 24]. Extracts of kokum were found to have antimicrobial activity against *Micrococcus aureus*, *Bacillus megaterium*, *Micrococcus luteus*, *Salmonella typhimurium*, *Pseudomonas aeruginosa*, *E. coli*, *Bacillus subtilis*, *Enterobacter aerogenes*, and *Staphylococcus aureus* [25,26].

Clove (*Syzygium aromaticum*), a native of the Maluku Islands in Indonesia, is one of the most valuable spices that has been used for centuries as a food preservative and other medicinal purposes. This spice is currently grown in several countries including Indonesia, India, Malaysia, Sri Lanka, Madagascar, Tanzania, and Brazil [27]. Clove has high antioxidant activity which is mostly due to its flavonoid (e.g., quercetin) and essential oil (e.g., eugenol) content [28-30]. Clove also has potential antihyperglycemic properties through inhibition of carbohydrate digestive enzymes such as α-amylase and α-glucosidase, which is a common therapeutic target to manage chronic hyperglycemia [31,32]. Clove (flower buds) and clove essential oils (e.g., eugenol) have shown antimicrobial activity against different gram-positive (*Staphylococcus, Listeria,* and *Bacillus*) and gram-negative (*Enterobacter*, *Shigella, E. coli, Salmonella, Klebsiella,* and *Pseudomonas*) bacterial pathogens, thereby making them valuable natural preservatives with potential for food safety applications [33-37]. Similarly, garlic (*Allium sativum*) is another example of a botanical with phytopharmacological properties that include antioxidant, antihypertension, antidiabetic, and antimicrobial activity [38-43]. The bioactivity of garlic is mostly due to the presence of organosulfur compounds such as allicin and S-allylcysteine which are present at a concentration of around 2.3 % [44-46].

Based on these wider antimicrobial and health protective functional properties of the above botanicals, the goal of this study was to screen different forms of these select food aligned botanicals (powder, slice, or pickle), for their antimicrobial activity against strains of bacterial pathogens (*E. coli*, *Listeria*, and *Salmonella*) that are associated with foodborne illnesses. Also, the total soluble phenolic content, phenolic profile, antioxidant activity, and enzyme inhibitory activity against type 2 diabetes-relevant α-amylase and α-glucosidase and hypertensive-relevant angiotensin-I-converting enzyme (ACE), were also investigated to evaluate their dual functional food safety and NCD countering benefits. The results of this study will help determine the food safety-relevant and human health protective functional benefits of these food-based botanicals which can be utilized in future dietary strategies to help counter foodborne illnesses caused due to microbial contamination, as well as mitigate and manage the prevalence of type 2 diabetes and chronic hypertension associated with diet-linked non-communicable chronic diseases.

2. Materials and Methods

2.1. Chemicals used

Other than mentioned, all chemicals and enzymes were purchased from Sigma Chemical Co (St. Louis, MO).

2.2. Samples used

Clove (flower buds), amla (powder, slice, and pickle), kokum (dried slices), and garlic (slice and pickle) were purchased from a local Indian grocery store of Fargo (North Dakota, USA). The clove and kokum samples were ground using a coffee blender to obtain a coarse

powder while the amla, kokum, and garlic samples (slice and pickle) were chopped into smaller pieces before extraction.

2.3. Preparation of extracts

The extraction of all samples was done using hot water. For the clove, amla, and kokum samples (powder and pickle), 10 g of the sample was used in the hot water extraction protocol, while for the rest of the samples- amla, kokum, and garlic (slices), 25 g of the sample was used. For the hot water extraction protocol, 10 g or 25 g of the respective samples were added to 50 mL of boiling water (100 °C) and boiled for 15 min after which the samples were cooled down to room temperature and centrifuged at 8,500 rpm for 15 min. The supernatant was collected and re-centrifuged at 8,500 rpm for 15 min and the extracts were stored at -20 °C. For the antimicrobial assay, the frozen extracts were thawed at room temperature and filter-sterilized using 0.22 µm syringe filters (Millipore Corp, MA, USA) prior to the assay. The amla slice, amla pickle, garlic slice and garlic pickle extracts were analyzed on a fresh weight (FW) basis while the clove, amla powder, kokum slice and kokum powder extracts were analyzed on a dry weight (DW) basis.

2.4. Bacterial strains used

The *Salmonella* strains tested in this study were *Salmonella enterica* subsp. *enterica* serovar Enteritidis (ATCC BAA-1045), *S. enterica* subsp. *enterica* serovar Typhimurium (FSL R8-0865), *S. enterica* subsp. *enterica* serovar Montevideo (FSL R8-3417), *S. enterica* subsp. *enterica* serovar Stanley (FSL R8-3511), and *S. enterica* subsp. *enterica* serovar Saintpaul (FSL R8-3582). The *Listeria* strains used in this study were *Listeria monocytogenes* serovar 1/2a (10403S), *L. monocytogenes* serovar 1/2b (FSL J1-0194), *L. monocytogenes* serovar 1/2a (FSL

F2-0515), and *L. monocytogenes* serovar 4b (H7858). The *E. coli* strains used in this study were *E. coli* serovar O157:H7 (EDL-933) and *E. coli* serovar O26:H11 (TW07936). The *Salmonella* and *Listeria* stocks were stored at -80°C in brain heart infusion broth (Oxoid, Basingstoke, UK) containing 25 % glycerol. The *E. coli* stocks were stored at -80 °C in LB broth, Miller (Luria-Bertani) (Difco, Becton, Dickinson and Company, Sparks, MD) containing 25 % glycerol. The *Salmonella* and *Listeria* strains were obtained from the Food Safety Laboratory at Cornell University, except for *Salmonella enterica* subsp. *enterica* serovar Enteritidis (ATCC BAA-1045) which was obtained from the American Type Culture Collection (Manassas, VA). The *E. coli* strains were obtained from the Thomas S. Whittam STEC Center at Michigan State University.

2.5. Total soluble phenolic (TSP) content

The TSP content of the extracts was determined using the Folin-Ciocalteu method based on a protocol as described previously [47]. For this assay, the extracts were diluted in water at 1:20 dilution and 0.5 mL aliquots of the diluted extracts were taken into respective glass tubes after which 1 mL of 95% ethanol, 0.5 mL of 50 % (v/v) Folin-Ciocalteu reagent, and 1 mL of 5 % sodium carbonate were added sequentially to each tube. Due to higher ascorbic acid content of the amla, which interferes with the Folin-Ciocalteu reagent, we have used multiple dilutions of the samples to avoid overestimation of total soluble phenolic content. The tubes were then mixed using a vortex machine and incubated for 60 min under dark conditions. The absorbance values in each tube were measured at 725 nm with a UV-visible spectrophotometer (Genesys 10S UV-VIS spectrophotometer, Thermo Scientific, NY, USA). Using a standard curve of different concentrations of gallic acid in 95 % ethanol, the absorbance values of the extracts were

converted, and the TSP content was expressed in milligram gallic acid equivalents per gram fresh weight or dry weight (mg GAE/g FW or DW).

2.6. Phenolic profile

The profile of the phenolic compounds was determined using the high-performance liquid chromatography (HPLC) assay method. The extracts were centrifuged at 13,500 rpm for 5 min after which 5 μL of the supernatant were injected using an Agilent ALS 1200 auto-extractor into an Agilent 1260 series (Agilent Technologies, Palo Alto, CA) HPLC equipped with a D1100 CE diode array detector. The solvents used for gradient elution were 10 mM phosphoric acid (pH 2.5) and 100 % methanol. The methanol concentration was increased to 60 % for the first 8 min, then to 100 % over the next 7 min, then decreased to 0 % for the next 3 min and was maintained for 7 min with a total run time of 25 min per injected sample run. The analytical column used was Agilent Zorbax SB-C18, 250 − 4.6 mm i.d., with packing material of 5 μm particle size at a flow rate of 0.7 mL/min at room temperature. The absorbance values were recorded at 214 nm, 230 nm, 260 nm, and 306 nm and the chromatogram was integrated using Agilent Chem station enhanced integrator. Pure standards of benzoic acid, gallic acid, protocatechuic acid, ellagic acid, cinnamic acid, dihydroxybenzoic acid, p-coumaric acid, rutin, and catechin in 100 % methanol were used to calibrate the respective standard curves and retention times. The phenolic compounds detected in the extracts were expressed in microgram per gram fresh weight or dry weight (μg/g FW or DW).

2.7. Antimicrobial activity

The antimicrobial activity of the extracts was measured using the broth microdilution method based on the protocol as described by the Clinical and Laboratory Standards Institute

[48]. A single colony of each bacterial strain was inoculated into 15 mL centrifuge tubes containing 10 mL Mueller-Hinton (MH) broth and the tubes were incubated overnight at 37 °C. The overnight culture was centrifuged, and the bacterial pellet was resuspended in 10 mL phosphate-buffered saline (PBS) (VWR). The turbidity of each culture was adjusted with PBS to a 0.5 McFarland standard with the help of a 0.5 McFarland standard reference solution (Remel, Thermo Fisher Scientific, Waltham, MA) and the turbidity was confirmed using a UV-visible spectrophotometer (SmartSpec3000, Bio-Rad, Hercules, CA). The adjusted cultures were then diluted in PBS at 1:20 dilution and used as the inoculum in the broth microdilution assay. A two-fold serial dilution of each filter-sterilized extract was done in microtiter plates using MH broth as the dilutant. For the control wells, sterile water was used instead of the extract. Around 10 µL of the adjusted bacterial inoculum were added to the respective wells and the microtiter plates were sealed with a plastic film and incubated for 16 h at 37 °C in a microplate reader (Bio Tek Instruments, Agilent Technologies). At every 15-min interval the microtiter plate was shaken for 5 seconds followed by a measurement of the absorbance of the wells at 600 nm. The absorbance values were plotted on a graph to get the growth curves and the minimal inhibitory concentration (MIC) of the extracts was expressed in mg GAE/g FW or DW.

2.8. Antioxidant activity

The antioxidant activity of the extracts was measured by their scavenging activity against the free radicals 2, 2-Dipheny-1-Picryl Hydrazyl (DPPH) (D9132-5G, Sigma-Aldrich), and 2, 2-Azino-bis-(3-ethylbenzthiazoline-6-sulfonic acid) (ABTS) (A1888-5G, Sigma-Aldrich) respectively. The DPPH scavenging assay was based on a protocol as described earlier [49] in which 0.25 mL of the extracts were added to 1.25 mL of 60 mM DPPH prepared in 95% ethanol while the controls had 0.25 mL of 95 % ethanol instead of the extract. After 5 min of incubation,

the extracts and their corresponding controls were centrifuged at 13,000 rpm for 1 min and the absorbance values of the supernatants were measured at 517 nm using a UV-visible spectrophotometer (Genesys 10S UV-VIS spectrophotometer, Thermo Scientific, NY). The ABTS scavenging assay was based on a protocol as described earlier [50] in which 0.05 mL of the extracts was added to 1 mL of ABTS prepared in 95 % ethanol while the controls had 0.05 mL of 95 % ethanol instead of the extract. After 2 min of incubation, the extracts and their controls were centrifuged at 13,000 rpm for 1 min and the absorbance values of the supernatant were measured at 734 nm with a UV-visible spectrophotometer (Genesys 10S UV-VIS spectrophotometer, Thermo Scientific, NY). The absorbance values from the DPPH and ABTS radical scavenging assays were used to calculate the percentage of antioxidant activity for each extract using the following formula:

$$\% \text{ Antioxidant activity} = \frac{\text{Control}^{\text{absorbance}} - \text{Extract}^{\text{absorbance}}}{\text{Control}^{\text{absorbance}}} \times 100$$

2.9. Alpha-amylase enzyme inhibitory activity

The α-amylase enzyme inhibitory activity of the extracts was measured based on a protocol as described earlier [49] and the activity was measured in a dose-dependent manner at undiluted, half, and one-fifth dilutions of the extracts. The extracts were diluted using distilled water and 500 µL of undiluted and diluted extracts were added to respective glass tubes, while the control tubes had 500 µL of 0.1M sodium phosphate buffer (containing 0.006M sodium chloride at pH 6.9) instead of the extract. Additionally, each extract had a corresponding blank tube containing 500 µL of the extract and 500 µL of the buffer instead of the enzyme. Then 500 µL of porcine pancreatic α-amylase (0.5 mg/mL buffer) (EC 3.2.1.1, purchased from Sigma

Chemical Co, St Louis, MO) was added to the extract and control tubes and incubated for 10 min at 25 °C. After incubation 500 μL of the substrate (1% starch in buffer) was added to all the tubes and incubated again for 10 min at 25 °C. Then 1 mL of 3, 5 dinitro salicylic acid was added and the tubes were incubated in a boiling water bath for 10 min to stop the reaction. After removing from the water bath and cooling down to room temperature, 10 mL of distilled water was added to all the tubes to ensure that the absorbance values in the control tubes ranged between 1.0 and 1.2, and the absorbance of all the tubes was measured at 540 nm using a UV-visible spectrophotometer (Genesys 10S UV-VIS spectrophotometer, Thermo Scientific, NY). The absorbance values were then used to calculate the percentage of enzyme inhibitory activity of the extracts using the following formula:

$$\% \text{ Inhibition} = \frac{\text{Control}^{\text{absorbance}} - (\text{Extract}^{\text{absorbance}} - \text{Extract blank}^{\text{absorbance}})}{\text{Control}^{\text{absorbance}}} \times 100$$

Using a standard curve of different concentrations of acarbose in distilled water, the percentages of α-amylase inhibitory activity obtained from the enzyme inhibition assay were expressed as millimolar equivalents of acarbose (mM AE).

2.10. Alpha-glucosidase enzyme inhibitory activity

The α-glucosidase enzyme inhibitory activity of the extracts was measured based on a protocol as described earlier [49] and the activity was measured in a dose-dependent manner at undiluted, half and one-fifth dilutions of the extracts. The extracts were diluted with 0.1M potassium phosphate buffer (pH 6.9) in 96 well microtiter plates in which 50 μL, 25 μL, and 10 μL of each extract were pipetted and the final volume made up to 50 μL by the addition of potassium phosphate buffer. Each extract had a corresponding control of 50 μL buffer instead of

the sample and the volume in all the wells was made up to a final volume of 100 µL by the addition of 50 µL of the buffer. Then 100 µL of buffer containing yeast α-glucosidase enzyme (1 U/mL) (EC 3.2.1.20, purchased from Sigma Chemical Co, St. Louis, MO) was added to each well and incubated for 10 min at 25 °C, after which 50 µL of the substrate, 5 mM p-nitrophenyl-α-D- glucopyranoside solution (prepared in buffer) was added to each well followed by 5-min incubation at 25 °C. The absorbance of all the wells was measured at 405 nm using a microplate reader (Thermomax, Molecular device Co., VA) at the 0- and 5-min time points of the 5-min incubation period, and the absorbance values were used to calculate the percentage of enzyme inhibitory activity using the following formula:

$$\% \text{ Inhibition} = \frac{(\text{Control}^{abs}\ 5\ \text{min} - \text{Control}^{abs}\ 0\ \text{min}) - (\text{Extract}^{abs}\ 5\ \text{min} - \text{Extract}^{abs}\ 0\ \text{min})}{(\text{Control}^{abs}\ 5\ \text{min} - \text{Control}^{abs}\ 0\ \text{min})} \times 100$$

Using a standard curve of different concentrations of Acarbose in distilled water, the percentages of α-glucosidase inhibitory activity obtained from the enzyme inhibition assay were expressed as millimolar equivalents of acarbose (mM AE).

2.11. Angiotensin-I-converting enzyme (ACE) inhibitory activity

The ACE inhibitory activity of the extracts was measured based on a protocol as described earlier [49] and the activity was measured in a dose-dependent manner at undiluted, half, and one-fifth dilutions of the extracts. The extracts were diluted using distilled water. The substrate used was hippuryl-histidyl-leucine (HHL) and the enzyme ACE was obtained from rabbit lung (EC 3.4.15.1). To 50 µL of the extracts, 200 µL of 1M NaCl-borate buffer (pH 8.3) containing 2mU of ACE was added and then incubated at room temperature for 10 minutes. For the blank tubes, 50 µL of distilled water and 200 µL of buffer were used instead of the extract

and enzyme. After this, 100 µL of 5mM HHL substrate (prepared in buffer) was added to all the tubes and incubated for one hour at 37 °C. The reaction was stopped by the addition of 150 µL of 0.5N HCl. The hippuric acid formed due to ACE activity was detected using the HPLC method for which, 5 µL of the reaction mixtures were injected using Agilent ALS 1200 autosampler into an Agilent 1260 series (Agilent Technologies, Palo Alto, CA) HPLC equipped with a D1100 CE diode array detector. The solvents used for the gradient were a combination of 10mM phosphoric acid (pH2.5) and 100% methanol. For the first 8 min, the methanol concentration was increased to 60% then to 100% for 5 min, and finally to 0% for the next 5 min and the total run time was 18 min for each sample. The analytical column used was Agilent Zorbax SB-C18, 250 × 4.6 mm i.d., with packing material of 5 µm particle size at a flow rate of 0.7 mL/min at room temperature. The absorbance was measured at 228 nm and the chromatogram was integrated using Agilent Chemstation (Agilent Technologies) enhanced integrator for detection of hippuric acid. A hippuric acid standard was used to calibrate the standard curve and retention time and the percentage of ACE inhibition was calculated using the formula:

$$\%\ \text{inhibition} = \frac{(\text{Control}^{\text{absorbance}} - \text{Extract}^{\text{absorbance}})}{(\text{Control}^{\text{absorbance}} - \text{Blank}^{\text{absorbance}})} \times 100$$

2.12. Statistical analysis

The complete in vitro analysis was repeated four times. Analysis at every time point from each experiment was carried out in triplicates except for the antimicrobial assay which was done in duplicate. The mean, standard error, and standard deviation were calculated from replicates within the experiments using Microsoft Excel XP. The data was analyzed with analysis of variance (ANOVA) using Statistical Analytical Software (SAS version 9.4; SAS Institute, Cary,

NC) and significant differences among extracts were determined by the Tukey's least mean square test at the 0.05 probability level.

3. Results and Discussion

3.1. Total soluble phenolic content and phenolic profile

The TSP content of different forms of select botanicals ranged from 0.7 to 132.6 mg GAE/g FW or DW and significant differences in TSP content were observed among the extracts ($p<0.05$) (Fig 1).

Fig 1. TSP content (mg GAE/g FW or DW) and antioxidant activity (mm TE) of the botanical extracts. Different lowercase letters indicate significant differences in TSP content among the extracts ($p<0.05$). Different uppercase letters indicate significant differences in antioxidant activity (ABTS and DPPH- based) among the extracts ($p<0.05$). The TSP content of amla slice, amla pickle, garlic slice and garlic pickle extracts were analyzed on a fresh weight (FW) basis while the clove, amla powder, kokum slice and kokum powder extracts were analyzed on a dry weight (DW) basis.

Amla powder had significantly higher TSP content at 132.6 mg GAE/g DW when compared to the rest of the extracts ($p<0.05$) while garlic slice had the lowest TSP content at 0.7 mg GAE/g FW (Fig 1). The TSP content of amla can vary depending upon the cultivar as well as the physical condition of the fruit (slices, juice, or powder) [51-53]. In the current study, although the TSP content of amla powder was significantly higher when compared to the other extracts ($p<0.05$), the content was still lower than what was reported in earlier studies [52,53]. The Folin-Ciocalteu (FC) reagent used in the estimation of TSP content can be affected by other

compounds such as ascorbic acid, sugars and organic acids that may be present in plant-based food matrix [54]. Therefore, the high ascorbic acid content in amla can potentially interfere with the accurate estimation of TSP content. In the current study, serial dilution of the extracts has been done to avoid overestimation of TSP content of amla. The phenolic profile and content of the extracts varied greatly, and statistical differences in the concentration of phenolic compounds detected were observed among the select botanical extracts ($p<0.05$) (Table 1).

Table 1. Phenolic profile of the botanical extracts expressed in microgram per gram fresh weight or dry weight (µg/g FW or DW).

Extracts	Gallic acid[a,b,c]	Ellagic acid[a,b,c]	Cinnamic acid[a,b,c]	Dihydroxy benzoic acid[a,b,c]	Benzoic acid[a,b,c]	p-coumaric acid[a,b,c]	Protocatechuic acid[a,b,c]	Rutin[a,b,c]	Catechin[a,b,c]
Clove powder	4.38 ± 0.6b	10.16 ± 1.3c	41.11 ± 1.2a	ND	ND	ND	ND	2.24 ± 0.2	13.67 ± 1.4b
Amla powder	4.51 ± 0.1b	133.38 ± 6.6a	5.94 ± 0.6b	ND	ND	ND	ND	ND	1.88 ± 0.0d
Amla slice	1.05 ± 0.1de	30.93 ± 1.6b	1.96 ± 0.1b	ND	0.15 ± 0.0	ND	ND	ND	1.53 ± 0.1d
Amla pickle	2.76 ± 0.2c	6.27 ± 0.3c	0.40 ± 0.0b	ND	ND	ND	ND	ND	1.35 ± 0.0d
Garlic slice	6.56 ± 0.1a	ND	ND	ND	ND	ND	ND	ND	3.42 ± 0.1d
Garlic pickle	1.67 ± 0.1d	ND	ND	2.42 ± 0.2c	ND	1.78 ± 0.1	ND	ND	2.93 ± 0.1d
Kokum powder	0.83 ± 0.0de	ND	ND	4.04 ± 0.0a	ND	ND	2.44 ± 0.0a	ND	24.01 ± 0.3a
Kokum slice	0.54 ± 0.0e	ND	ND	3.10 ± 0.0b	ND	ND	1.29 ± 0.0b	ND	10.18 ± 0.0c

ND- Not Detected

[a] Mean ± standard error

[b] Different letters in each column indicate significant differences among extracts ($p<0.05$).

[c] The phenolic concentration of the amla slice, amla pickle, garlic slice and garlic pickle extracts were analyzed on a fresh weight (FW) basis while the clove, amla powder, kokum slice and kokum powder extracts were analyzed on a dry weight (DW) basis.

The phenolic compounds detected were gallic acid (0.54 to 6.56 µg/g FW or DW), ellagic acid (6.27 to 133.38 µg/g FW or DW), cinnamic acid (0.40 to 41.11 µg/g FW or DW), dihydroxybenzoic acid (2.24 to 4.04 µg/g FW or DW), benzoic acid (0.15 µg/g FW or DW), p-coumaric acid (1.78 µg/g FW or DW), protocatechuic acid (1.29 to 2.44 µg/g FW or DW), rutin (2.24 µg/g FW or DW), and catechin (1.35 to 24.01 µg/g FW or DW). The garlic slice, amla powder, clove, and kokum powder extracts were found to have significantly higher concentrations of gallic acid, ellagic acid, cinnamic acid, and catechin, respectively ($p<0.05$) (Table 1). Benzoic acid, p-coumaric acid, and protocatechuic acid were detected only in the respective amla slice, garlic pickle, and kokum (powder and slice) extracts (Table 1). Overall, the TSP content and phenolic profile of this study indicated that the select botanicals are good sources of phenolic phytochemicals and the phenolic profile was found to vary among these botanicals. Furthermore, phytochemical-rich fruits such as amla or kokum can be incorporated as fresh slices, powder, or pickles, as dietary strategies aimed at improving the health protective benefits.

3.2. Antimicrobial activity

The antimicrobial activity of the botanical extracts was measured against serovars of *Salmonella*, *Listeria* and *E. coli* and the minimal inhibitory concentration (MIC) was expressed in mg GAE/g FW or DW. The MIC of the extracts against *Salmonella*, *Listeria*, and *E. coli* serovars ranged from 0.25 to 22.00 mg GAE/g FW or DW, 0.12 to 11.00 mg GAE/g FW or DW, and 0.25 to 22.00 mg GAE/g FW or DW, respectively, and statistical differences in MIC was observed among all the botanical extracts for all the microorganisms that were tested ($p<0.05$) (Table 2).

Table 2. Minimal inhibitory concentration (MIC) of the botanical extracts expressed in milligram gallic acid equivalents per gram fresh weight or dry weight (mg GAE/g FW or DW).

**Extracts**	**Minimal inhibitory concentration[a,b]**										
	Salmonella					*Listeria*				*E. coli*	
	S. enterica subsp. *enterica* serovar Enteritidis	*S. enterica* subsp. *enterica* serovar Typhimurium	*S. enterica* subsp. *enterica* serovar Montevideo	*S. enterica* subsp. *enterica* serovar Stanley	*S. enterica* subsp. *enterica* serovar Saintpaul	*L. monocytogenes* serovar 1/2a	*L. monocytogenes* serovar 1/2b	*L. monocytogenes* serovar 1/2a	*L. monocytogenes* serovar 4b	*E. coli* serovar O157:H7	*E. coli* serovar O26:H11
Clove powder	22.00 ± 0.0a	22.00 ± 0.0a	22.00 ± 0.0a	44.00 ± 0.0a	44.00 ± 0.0a	11.00 ± 0.0a	NA	NA	NA	22.00 ± 0.0a	22.00 ± 0.0a
Amla powder	8.29 ± 0.0c	8.29 ± 0.0c	8.29 ± 0.0b	33.16 ± 0.0b	16.58 ± 0.0c	4.15 ± 0.0c	4.15 ± 0.0b	4.15 ± 0.0b	4.15 ± 0.0b	8.29 ± 0.0c	8.29 ± 0.0c
Amla slice	9.54 ± 0.0b	9.54 ± 0.0b	7.16 ± 2.4b	19.09 ± 0.0c	19.09 ± 0.0b	4.77 ± 0.0b	4.77 ± 0.0a	4.77 ± 0.0a	9.54 ± 0.0a	9.54 ± 0.0b	9.54 ± 0.0b
Amla pickle	6.67 ± 0.0d	6.67 ± 0.0d	6.67 ± 0.0b	6.67 ± 0.0d	6.67 ± 0.0d	3.33 ± 0.0d	3.33 ± 0.0c	3.33 ± 0.0c	3.33 ± 0.0c	6.67 ± 0.0d	6.67 ± 0.0d
Garlic slice	0.36 ± 0.0g	NA	NA	NA	NA	NA	NA	NA	NA	NA	NA
Garlic pickle	0.85 ± 0.0f	0.85 ± 0.0f	NA	NA	NA	NA	NA	NA	NA	0.85 ± 0.0e	0.85 ± 0.0e
Kokum powder	1.65 ± 0.0e	1.65 ± 0.0e	3.30 ± 0.0bc	3.30 ± 0.0e	3.30 ± 0.0e	0.82 ± 0.0e	0.41 ± 0.0d	0.41 ± 0.0d	0.41 ± 0.0d	0.82 ± 0.0f	0.82 ± 0.0f
Kokum slice	0.25 ± 0.0h	0.25 ± 0.0g	0.50 ± 0.0c	0.50 ± 0.0f	0.50 ± 0.0f	0.12 ± 0.0f	0.12 ± 0.0e	0.12 ± 0.0e	0.12 ± 0.0e	0.25 ± 0.0g	0.25 ± 0.0g

NA- No Activity

[a] Mean ± standard error

[b] Different lowercase letters in each column indicate significant differences in MIC among the extracts (p<0.05).

Among the *Salmonella* serovars (Enteritidis, Typhimurium, Montevideo, Stanley, and Saintpaul), extracts of kokum slice had lower MIC at 0.25 and 0.50 mg GAE/g DW ($p<0.05$), while extracts of clove had the highest MIC at 22.00 and 44.00 mg GAE/g DW (Table 2). No antimicrobial activity was detected for the garlic extracts (slice and pickle) against the *Salmonella* serovars Montevideo, Stanley, and Saintpaul. Instead, the garlic extracts enhanced the growth of these serovars when compared to the control (data not shown). Among the *Listeria* serovars (1/2a, 1/2b, and 4b), extracts of kokum slice had lower MIC at 0.12 mg GAE/g DW ($p<0.05$), while extracts of clove had the highest MIC at 11.00 mg GAE/g DW (Table 2). No antimicrobial activity was detected for the clove and garlic (slice and pickle) extracts against the *Listeria* serovars 1/2a, 1/2b, and 4b. Among the *E. coli* serovars (O157:H7 and O26:H11), extracts of kokum slice had lower MIC at 0.25 mg GAE/g DW ($p<0.05$), while extracts of clove had the highest MIC at 22.00 mg GAE/g DW (Table 2). No antimicrobial activity was detected for the garlic slice and pickle extracts against most of the food-borne disease associated bacterial pathogens that were tested. Overall, the *Listeria* serovars were more susceptible towards the antimicrobial activity of the select botanicals as evident by the lower MIC values when compared to *Salmonella* or *E. coli*. The mechanism of antimicrobial activity of phenolic phytochemicals is due to their ability to permeabilize the microbial cell membrane, inhibit DNA or protein synthesis, inhibit microbial metabolic activity, as well as chelate compounds required for microbial growth [55-58]. The antimicrobial activity observed in the current study clearly suggests that these select botanicals, especially amla and kokum, with high baseline phenolic content are good bioactive ingredients to counter bacterial contamination of other plant-based foods. This in the long term can potentially help mitigate the prevalence of foodborne illnesses.

3.3. Antioxidant activity

The antioxidant activity of the extracts was measured via their ABTS and DPPH radical scavenging activity, and the activity was expressed in millimolar Trolox equivalents (mM TE). The ABTS and DPPH scavenging activity ranged from 0.49 to 0.52 mM TE/mL and 0.09 to 0.39 mM TE, respectively, and statistical differences in radical scavenging activity were observed among the extracts ($p<0.05$) (Fig 1). Amla slice and amla pickle extracts had higher ABTS scavenging activity at 0.52 mM TE ($p<0.05$), while garlic slice had the lowest ABTS scavenging at 0.49 mM TE. Similarly, amla slice extract had higher DPPH scavenging activity at 0.39 mM TE when compared to the rest of the botanical extracts ($p<0.05$), while garlic slice had the lowest DPPH scavenging activity at 0.09 mM TE (Fig 1). The select botanicals had higher ABTS scavenging activity when compared to DPPH scavenging activity, which could be due to the difference in the chemical nature of these synthetic radicals, as DPPH being more stable than ABTS, requires stronger antioxidant activity. In an earlier study, amla extracts showed high DPPH radical scavenging activity at 92.1% [59]. In another study, ice containing kokum was found to improve the oxidative stability and shelf life of chilled Indian mackerel (*Rastrelliger kanagurta*) [60]. These results indicate that amla and kokum fruit can be incorporated in dietary strategies as slices, powder, or pickle, due to their phytochemical-linked antioxidant activity which can offer chronic oxidative stress protective benefits relevant for the management of common NCDs.

3.4. Alpha-amylase and α-glucosidase enzyme inhibitory activity

The α-amylase and α-glucosidase inhibitory activity of the extracts was measured in a dose-dependent manner using undiluted, half-, and one-fifth dilutions of the extracts, and the inhibitory activity was expressed in millimolar acarbose equivalents (mM AE). The α-amylase inhibitory activity of the undiluted, half-, and one-fifth diluted extracts ranged from 0.03 to 0.19

mM AE, 0.00 to 0.19 mM AE, and 0.00 to 0.18 mM AE, respectively, and statistical differences in α-amylase inhibitory activity were observed among the undiluted, half-, and one-fifth dilutions of the extracts ($p<0.05$) (Table 3).

Table 3. Alpha-amylase and α-glucosidase enzyme inhibitory activity of the botanical extracts expressed in millimolar acarbose equivalents (mM AE).

Extracts	α-amylase inhibitory activity[a,b]			α-glucosidase inhibitory activity[a,b]		
	Undiluted	Half-diluted	One-fifth-diluted	Undiluted	Half-diluted	One-fifth-diluted
Clove powder	0.18 ± 0.01ab	0.17 ± 0.00ab	0.06 ± 0.00c	1.61 ± 0.01a	1.61 ± 0.01a	1.52 ± 0.02b
Amla powder	0.19 ± 0.00a	0.18 ± 0.01ab	0.17 ± 0.00a	1.62 ± 0.02a	1.61 ± 0.00a	1.59 ± 0.00a
Amla slice	0.17 ± 0.01ab	0.17 ± 0.00ab	0.16 ± 0.00b	1.62 ± 0.00a	1.62 ± 0.00a	1.54 ± 0.01b
Amla pickle	0.15 ± 0.01c	0.14 ± 0.01c	0.03 ± 0.00d	1.44 ± 0.03b	1.22 ± 0.02b	1.20 ± 0.01c
Garlic slice	0.03 ± 0.00d	0.005 ± 0.00d	NA	0.24 ± 0.01d	0.04 ± 0.03d	NA
Garlic pickle	0.16 ± 0.01bc	0.12 ± 0.01c	0.03 ± 0.00d	1.17 ± 0.01c	0.80 ± 0.01c	0.35 ± 0.01d
Kokum powder	0.19 ± 0.00a	0.19 ± 0.00a	0.18 ± 0.00a	1.61 ± 0.00a	1.61 ± 0.01a	1.61 ± 0.00a
Kokum slice	0.19 ± 0.00a	0.19 ± 0.00a	0.18 ± 0.00a	1.59 ± 0.01a	1.59 ± 0.02a	1.52 ± 0.01b

NA- No Activity.

[a] Mean ± standard error.

[b] Different letters in each column indicate significant differences among extracts ($p<0.05$).

Among the undiluted extracts, the clove, kokum and amla extracts (slice and powder) had higher α-amylase inhibitory activity ranging from 0.18 to 0.19 mM AE ($p<0.05$), while garlic slice had the lowest α-amylase inhibitory activity at 0.03 mM AE. The same trend was observed for the half-diluted and one-fifth-diluted extracts (Table 3). The α-glucosidase inhibitory activity

of the undiluted, half-, and one-fifth diluted extracts ranged from 0.24 to 1.62 mM AE, 0.04 to 1.62 mM AE, and 0.00 to 1.61 mM AE, respectively, and statistical differences in α-glucosidase inhibitory activity were observed among the undiluted, half-, and one-fifth-diluted extracts (p<0.05) (Table 3). Among the undiluted extracts, the clove, kokum and amla extracts (slice and powder) had higher α-glucosidase inhibitory activity ranging from 1.59 to 1.62 mM AE (p<0.05), while garlic slice had the lowest α-glucosidase inhibitory activity at 0.24 mM AE. A similar trend was observed for the half-and one-fifth dilutions of the extracts. Interestingly, extracts of amla and kokum (slices and powder) had the same level of α-amylase and α-glucosidase inhibitory activity, even at one-fifth dilutions of the extracts.

The amla and kokum (slice or powder) analyzed in the current study were found to display high α-amylase and α-glucosidase inhibitory activity even at lower dilutions of the extracts, thereby indicating their potent antihyperglycemic activity. In an earlier study, phenolic-rich fractions of kokum were found to have α-amylase inhibitory activity with IC_{50} values ranging from 349.7 to 980.0 µg/mL [61]. In another study, extracts of amla at the highest concentration were found to have α-amylase and α-glucosidase inhibitory activity at 84.15% and 93.9% respectively [59]. Grape seed and tea extracts were found to have *in vitro* α-amylase and α-glucosidase inhibitory activity and the activity was attributed to the catechin content [62]. In the current study, catechin, a flavonoid compound with potential anti-hyperglycemic activity, was detected in all the botanical extracts. The results of the current *in vitro* assay model-based screening study indicate that amla and kokum (slice or powder) with high carbohydrate-digestive enzyme inhibitory potential can be incorporated into dietary strategies aimed at the management of chronic hyperglycemia, a common risk factor associated with type 2 diabetes. However, future

clinical studies are required to further validate the anti-diabetic potential of amla and kokum and for its value-added integration in health targeted dietary and therapeutic applications.

3.5. Angiotensin-I-converting enzyme (ACE) inhibitory activity

The ACE inhibitory activity was measured in a dose-dependent manner using undiluted, half-, and one-fifth dilutions of the extracts, and the inhibitory activity was expressed in percentages (%). The ACE inhibitory activity of the undiluted, half-diluted, and one-fifth diluted extracts ranged from 90.1 to 100 %, 45.9 to 100 %, and 0 to 100 %, respectively, and statistical differences in ACE inhibitory activity were observed among the undiluted, half-, and one-fifth-diluted extracts (p<0.05) (Fig 2).

Fig 2. Angiotensin-I-converting enzyme inhibitory activity of the botanical extracts expressed as percentages (%). Different uppercase letters represent significant differences among the extracts (p<0.05).

Among the undiluted extracts, amla and kokum extracts (powder and slice) had significantly higher ACE inhibitory activity at 100% (p<0.05), while extracts of clove, amla pickle, and garlic (slice and pickle) had lower baseline ACE inhibitory activity ranging from 90.1 to 93.3%. (Fig 2). A similar trend was observed for the half-, and one-fifth diluted extracts. In the current study, the ACE inhibitory activity of the amla powder and kokum (powder and slice) extracts was found to be high even at one-fifth dilutions of the extracts, thereby indicating their potent antihypertensive activity. In a clinical study, an eight-week combination therapy of amla with antihypertensive drugs was found to significantly reduce the systolic blood pressure and diastolic blood pressure in patients with hypertension when compared to control (placebo) group [63]. In the current study, the *in vitro* ACE inhibition results indicates that amla and kokum, in

addition to the other select botanicals, can be incorporated in dietary support strategies as slices or powder to counter chronic hypertension commonly associated with type 2 diabetes and other NCDs.

4. Conclusion

The screening of underutilized botanicals and plant-based foods including fruits, spices and other food ingredients is an important first step necessary for the selection and utilization these phytochemical-rich food-based botanicals with associated dual functional food safety-relevant and NCD health protective benefits. Under-utilized or indigenous fruits such as amla or kokum in different forms (slice, powder, or pickle) display NCD health protective properties via their antioxidant, antihyperglycemic, and antihypertensive activity, and hence can be potentially incorporated in dietary strategies to counter chronic inflammation, hyperglycemia, and hypertension. In addition to their health protective benefits, these phytochemical-rich fruits and botanicals with dual functional antimicrobial property also have food safety-relevant benefits, specifically to counter foodborne illness related bacterial pathogens such as *Salmonella*, *Listeria*, and *E. coli*. The formulation of different forms of amla and kokum (slice, powder, or pickle) with other bioactive plant-based foods can potentially reduce the risk of microbial contamination with these bacterial pathogens and help manage the burden of foodborne illnesses. Future studies can focus on formulation or dual functional synergy strategies involving amla, kokum, and clove with other bioactive plant-based foods to improve phenolic phytochemical-linked functional qualities for wider food safety and human health benefits, including the potential management of NCDs as indicated in this study.

Acknowledgments

The authors would like to thank Dr. Teresa Bergholz, Michigan State University, for her assistance in obtaining the bacterial strains. The authors would also like to thank Scott Hoselton, North Dakota State University, for his feedback regarding the antimicrobial assay.

References

1. Khan KH. Roles of *Emblica officinalis* in medicine-A review. Bot Res Int. 2009; 2(2): 218-228.

2. Akhtar MS, Ramzan A, Ali A, Ahmad M. Effect of Amla fruit (*Emblica officinalis* Gaertn.) on blood glucose and lipid profile of normal subjects and type 2 diabetic patients. Int J Food Sci Nutr. 2011; 62(6): 609-616. doi:10.3109/09637486.2011.560565

3. Baliga MS, Shivashankara AR, Thilakchand KR, Baliga-Rao MP, Palatty PL, George T, et al. Hepatoprotective effects of the Indian Gooseberry (*Emblica officinalis* Gaertn): a revisit. In: Watson RR, Preedy VR, editors. Dietary interventions in liver disease. Cambridge: Academic press; 2019. pp. 193-201. doi:10.1016/B978-0-12-814466-4.00016-1

4. Bhandari PR, Kamdod MA. *Emblica officinalis* (Amla): A review of potential therapeutic applications. Int J Green Pharm. 2012; 6(4): 257-269. Available from: http://www.greenpharmacy.info/index.php/ijgp/article/view/272

5. D'souza JJ, D'souza PP, Fazal F, Kumar A, Bhat HP, Baliga MS. Anti-diabetic effects of the Indian indigenous fruit *Emblica officinalis* Gaertn: active constituents and modes of action. Food Funct. 2014; 5(4): 635-644. doi:10.1039/C3FO60366K

6. Khopde SM, Priyadarsini KI, Mohan H, Gawandi VB, Satav JG, Yakhmi JV, et al. Characterizing the antioxidant activity of amla (*Phyllanthus emblica*) extract. Curr Sci. 2001; 81: 185-190. Available from: https://www.jstor.org/stable/24106256

7. Kumar GS, Nayaka H, Dharmesh SM, Salimath PV. Free and bound phenolic antioxidants in amla (*Emblica officinalis)* and turmeric (*Curcuma longa).* J Food Compost Anal. 2006; 19(5): 446-452. doi: 10.1016/j.jfca.2005.12.015

8. Sonkar N, Rajoriya D, Chetana R, Murthy KV. Effect of cultivars, pretreatment and drying on physicochemical properties of Amla (*Emblica officinalis*) gratings. J Food Sci Technol. 2020; 57: 980-992. doi:10.1007/s13197-019-04131-8

9. Gautam A, Shukla S. *Emblica officinalis* (Amla) leaf extract potentiates antibacterial activity of some antibiotics. J Pharmacogn Phytochem. 2017; 6(2): 233-236. Available from: https://www.phytojournal.com/archives/2017/vol6issue2/PartE/6-2-40-684.pdf

10. Saeed S, Tariq P. Antimicrobial activities of *Emblica officinalis* and *Coriandrum sativum* against gram positive bacteria and Candida albicans. Pak J Bot. 2007; 39(3): 913-917.

11. Singh SN, Moses A, David A. Antimicrobial Activity of *Emblica officinalis* extracts against selected bacterial pathogens. Int J Basic and Appl Res. 2019; 9(1):325-30.

12. Tyagi A, Singh KP. Study on the evaluation of phenolic content and antimicrobial activities of Amla (*Emblica officinalis*) fruit. Int Res J Manag Sci Tech. 2014; 5(8): 3-13.

13. Vijayalakshmi S, Arunkumar V, Anju D, Gunasundari P, Moorthy P, Chandrasekharan AK. Comparative antimicrobial activities of *Emblica officinalis* and *Ocimum sanctum*. Anc Sci Life. 2007; 27(2): 1-6. PMID: 22557261

14. Alkandari D, Sarfraz H, Sidhu JS. Development of a functional food (pan bread) using amla fruit powder. J Food Sci Technol. 2019; 56: 2287-2295. doi:10.1007/s13197-019-03718-5

15. Alkandari D, Sarfraz H, Sidhu JS. Amla fruit powder addition influences objective color and instrumental texture of pan bread. J Food Sci Technol. 2019; 56:2750-2757. doi:10.1007/s13197-019-03766-x

16. Bhalerao PP, Mahale SA, Dhar R, Chakraborty S. Optimizing the formulation for a pomegranate-amla-muskmelon based mixed fruit beverage using sensory analysis and evaluating its thermal stability. LWT. 2020; 133:109907. doi: 10.1016/j.lwt.2020.109907

17. Patel AP, Bhagwat SR, Pawar MM, Prajapati KB, Chauhan HD, Makwana RB. Evaluation of *Emblica officinalis* fruit powder as a growth promoter in commercial broiler chickens. Vet World. 2016;9(2):207-210. doi: 10.14202/vetworld.2016.207-210

18. Zafar TA, Allafi AR, Alkandari D, Al-Othman A. Rheological characteristics of wheat–chickpea composite flour doughs and effect of Amla powder (*Phyllanthus emblica* L.) addition on the functional properties of bread. Food Sci Technol Int. 2021; 27(3):264-275. doi: 10.1177/1082013220950068

19. Ajjakana B, Nayak RP. Study of Antidepressant Activity of *Garcinia indica (*Kokum) Fruit Rind in Wistar Albino Rats. J Evol Med Dent Sci. 2021; 10(36):3077-3083.

20. Jagtap P, Bhise K, Prakya V. A phytopharmacological review on *Garcinia indica.* Int J Herb Med. 2015; 3(4):2-7. Available from: https://www.florajournal.com/vol3issue4/Oct2015/3-2-16.1.pdf

21. Lim SH, Lee HS, Lee CH, Choi CI. Pharmacological activity of *Garcinia indica* (Kokum): An updated review. Pharmaceuticals. 202; 14(12):1338. doi: 10.3390/ph14121338

22. Swami SB, Thakor NJ, Patil SC. Kokum (*Garcinia indica*) and its many functional components as related to the human health: a review. J Food Res Technol. 2014; 2(4):130-142.

23. Waghmare N, Shukla S, Kaur J. Kokum (*Garcinia indica*) a beneficial underutilised crop: A review. Think India Journal. 2019; 22(34):1354-1375. Available from: https://thinkindiaquarterly.org/index.php/think-india/article/view/18578

24. Singh P, Roy TK, Kanupriya C, Tripathi PC, Kumar P, Shivashankara KS. Evaluation of bioactive constituents of *Garcinia indica* (kokum) as a potential source of hydroxycitric acid, anthocyanin, and phenolic compounds. LWT. 2022; 156:112999. doi: 10.1016/j.lwt.2021.112999

25. Sutar RL, Mane SP, Ghosh JS. Antimicrobial activity of extracts of dried kokum (*Garcinia indica* C). Int Food Res J. 2012; 19(3):1207-1210. Available from: http://agris.upm.edu.my:8080/dspace/handle/0/12773

26. Varalakshmi KN, Sangeetha CG, Shabeena AN, Sunitha SR, Vapika J. Antimicrobial and cytotoxic effects of *Garcinia indica* fruit rind extract. Am-Eurasian J Agric Environ Sci. 2010; 7(6):652-656.

27. Cortés-Rojas DF, de Souza CR, Oliveira WP. Clove (*Syzygium aromaticum*): a precious spice. Asian Pac J Trop Biomed. 2014; 4(2):90-96. doi: 10.1016/S2221-1691(14)60215-X

28. Alfikri FN, Pujiarti R, Wibisono MG, Hardiyanto EB. Yield, quality, and antioxidant activity of clove (*Syzygium aromaticum* L.) bud oil at the different phenological stages in young and mature trees. Scientifica. 2020; 2020:1-8. doi: 10.1155/2020/9701701

29. Gülçin İ, Elmastaş M, Aboul-Enein HY. Antioxidant activity of clove oil–A powerful antioxidant source. Arab J Chem. 2012; 5(4):489-499. doi: 10.1016/j.arabjc.2010.09.016

30. Nassar MI, Gaara AH, El-Ghorab AH, Farrag A, Shen H, Huq E, Mabry TJ. Chemical constituents of clove (*Syzygium aromaticum,* Fam. Myrtaceae) and their antioxidant activity. Revista Latinoamericana de Química. 2007; 35(3):47-57. Available from: https://www.researchgate.net/publication/228357516

31. Adefegha SA, Oboh G, Adefegha OM, Boligon AA, Athayde ML. Antihyperglycemic, hypolipidemic, hepatoprotective and antioxidative effects of dietary clove (*Szyzgium aromaticum*) bud powder in a high□fat diet/streptozotocin□induced diabetes rat model. J Sci Food Agric. 2014; 94(13): 2726-2737.doi: 10.1002/jsfa.6617

32. Adefegha SA, Oboh G. In vitro inhibition activity of polyphenol-rich extracts from *Syzygium aromaticum* (L.) Merr. & Perry (Clove) buds against carbohydrate hydrolyzing enzymes linked to type 2 diabetes and Fe2+-induced lipid peroxidation in rat pancreas. Asian Pac J Trop Biomed. 2012; 2(10):774-781. doi: 10.1016/S2221-1691(12)60228-7

33. Arora DS, Kaur J. Antimicrobial activity of spices. Int J Antimicrob Agents. 1999; 12(3):257-262. doi: 10.1016/S0924-8579(99)00074-6

34. Ayoola GA, Lawore FM, Adelowotan T, Aibinu IE, Adenipekun E, Coker HA, Odugbemi TO. Chemical analysis and antimicrobial activity of the essential oil of *Syzigium*

aromaticum (clove). Afr J Microbiol Res. 2008; 2(7):162-166. Available from: https://www.researchgate.net/publication/237521165

35. Cava R, Nowak E, Taboada A, Marin-Iniesta F. Antimicrobial activity of clove and cinnamon essential oils against *Listeria monocytogenes* in pasteurized milk. J Food Prot. 2007; 70(12):2757-2763. doi: 10.4315/0362-028X-70.12.2757

36. Fu Y, Zu Y, Chen L, Shi X, Wang Z, Sun S, Efferth T. Antimicrobial activity of clove and rosemary essential oils alone and in combination. Phytother Res. 2007; 21(10):989-994. doi: 10.1002/ptr.2179

37. Mytle N, Anderson GL, Doyle MP, Smith MA. Antimicrobial activity of clove (*Syzgium aromaticum*) oil in inhibiting Listeria monocytogenes on chicken frankfurters. Food control. 2006; 17(2):102-107. doi: 10.1016/j.foodcont.2004.09.008

38. Ansary J, Forbes-Hernández TY, Gil E, Cianciosi D, Zhang J, Elexpuru-Zabaleta M, Simal-Gandara J, Giampieri F, Battino M. Potential health benefit of garlic based on human intervention studies: A brief overview. Antioxidants. 2020; 9(7):619. doi: 10.3390/antiox9070619

39. Matsutomo T. Potential benefits of garlic and other dietary supplements for the management of hypertension. Exp Ther Med 2020; 19(2):1479-1484. doi: 10.3892/etm.2019.8375

40. Nasir A, Fatma G, Neshat N, Ahmad MA. Pharmacological and therapeutic attributes of garlic (*Allium sativum* Linn.) with special reference to Unani medicine—A review. J Med Plants Stud. 2020; 8(3):6-9.

41. Parham S, Kharazi AZ, Bakhsheshi-Rad HR, Nur H, Ismail AF, Sharif S, Ramakrishna S, Berto F. Antioxidant, antimicrobial and antiviral properties of herbal materials. Antioxidants. 2020; 9(12):1309. doi: 10.3390/antiox9121309

42. Reiter J, Hübbers AM, Albrecht F, Leichert LI, Slusarenko AJ. Allicin, a natural antimicrobial defence substance from garlic, inhibits DNA gyrase activity in bacteria. Int. J Med Microbiol. 2020; 310(1):151359. doi: 10.1016/j.ijmm.2019.151359

43. Ried K. Garlic lowers blood pressure in hypertensive subjects, improves arterial stiffness and gut microbiota: A review and meta-analysis. Exp Ther Med. 2020; 19(2):1472-1478. doi: 10.3892/etm.2019.8374

44. Nakamoto M, Kunimura K, Suzuki JI, Kodera Y. Antimicrobial properties of hydrophobic compounds in garlic: Allicin, vinyldithiin, ajoene and diallyl polysulfides. Exp Ther Med. 2020; 19(2):1550-1553. doi: 10.3892/etm.2019.8388

45. Song H, Cui J, Mossine VV, Greenlief CM, Fritsche K, Sun GY, Gu Z. Bioactive components from garlic on brain resiliency against neuroinflammation and neurodegeneration. Exp Ther Med. 2020; 19(2):1554-1559. doi: 10.3892/etm.2019.8389

46. Zhang Y, Liu X, Ruan J, Zhuang X, Zhang X, Li Z. Phytochemicals of garlic: Promising candidates for cancer therapy. Biomed Pharmacother. 2020; 123:109730. doi: 10.1016/j.biopha.2019.109730

47. Shetty K, Curtis OF, Levin RE, Witkowsky R, Ang W. Prevention of Vitrification Aßociated with in vitro Shoot Culture of Oregano.(*Origanum vulgare)* by Pseudomonas spp. J Plant Physiol. 1995; 147(3-4):447-451. doi: 10.1016/S0176-1617(11)82181-4

48. CLSI. Methods for Dilution Antimicrobial Susceptibility Tests for Bacteria That Grow Aerobically; Approved Standard—Ninth Edition. CLSI document M07-A9. Wayne, PA: Clinical and Laboratory Standards Institute; 2012.

49. Kwon YI, Vattem DA, Shetty K. Evaluation of clonal herbs of Lamiaceae species for management of diabetes and hypertension. Asia Pac J Clin Nutr. 2006; 15(1):107-117. Available from: https://apjcn.nhri.org.tw/server/APJCN/15/1/107.pdf

50. Re R, Pellegrini N, Proteggente A, Pannala A, Yang M, Rice-Evans C. Antioxidant activity applying an improved ABTS radical cation decolorization assay. Free radical biology and medicine. 1999; 26(9-10):1231-1237. doi: 10.1016/S0891-5849(98)00315-3

51. Kaushal M, Sharma SK. Processing potential of newly introduced Amla cultivars grown in lower Himalayan Region of Himachal Pradesh. Int J Food Ferment Technol. 2012; 2(1):93-97.

52. Mishra P, Srivastava V, Verma D, Chauhan OP, Rai GK. Physico-chemical properties of Chakiya variety of Amla (*Emblica officinalis)* and effect of different dehydration methods on quality of powder. Afr J Food Sci. 2009; 3(10):303-6. Available from: https://academicjournals.org/journal/AJFS/article-full-text-pdf/59CE54520542

53. Tyagi A, Pal A. Physico-chemical properties of chakiya variety of amla (*Emblica officinalis*) and effect of different dehydration methods on quality of powder. World J Pharm Res. 2015; 4(6):1042-1048. Available from: https://wjpr.s3.ap-south-1.amazonaws.com/article_issue/1436597628.pdf

54. Lester GE, Lewers KS, Medina MB, Saftner RA. Comparative analysis of strawberry total phenolics via Fast Blue BB vs. Folin–Ciocalteu: Assay interference by ascorbic acid. J Food Compost Anal. 2012; 27(1):102-107. doi: 10.1016/j.jfca.2012.05.003

55. Christopher A, Sarkar D, Shetty K. Elicitation of stress-induced phenolic metabolites for antimicrobial applications against foodborne human bacterial pathogens. Antibiotics. 202; 10(2):109. doi: 10.3390/antibiotics10020109

56. Bouarab Chibane L, Degraeve P, Ferhout H, Bouajila J, Oulahal N. Plant antimicrobial polyphenols as potential natural food preservatives. J Sci Food Agric. 2019; 99(4):1457-1474. doi: 10.1002/jsfa.9357

57. Jayaraman P, Sakharkar KR, Sing LC, Chow VT, Sakharkar MK. Insights into antifolate activity of phytochemicals against *Pseudomonas aeruginosa*. J Drug Target. 2011; 19(3):179-188. doi: 10.3109/10611861003801867

58. Radulovic NS, Blagojevic PD, Stojanovic-Radic ZZ, Stojanovic NM. Antimicrobial plant metabolites: structural diversity and mechanism of action. Curr Med Chem. 2013; 20(7):932-952. doi: 10.2174/092986713805219136

59. Majeed M, Majeed S, Mundkur L, Nagabhushanam K, Arumugam S, Beede K, Ali F. Standardized *Emblica officinalis* fruit extract inhibited the activities of α□amylase, α□glucosidase, and dipeptidyl peptidase□4 and displayed antioxidant potential. J Sci Food Agric. 2020; 100(2):509-516. doi: 10.1002/jsfa.10020

60. Apang T, Xavier KM, Lekshmi M, Kannuchamy N, Layana P, Balange AK. Garcinia spp. extract incorporated icing medium as a natural preservative for shelf life enhancement of

chilled Indian mackerel (*Rastrelliger kanagurta*). LWT. 2020; 133:110086. doi: 10.1016/j.lwt.2020.110086

61. Munjal K, Ahmad S, Gupta A, Haye A, Amin S, Mir SR. Polyphenol-enriched fraction and the compounds isolated from Garcinia indica fruits ameliorate obesity through suppression of digestive enzymes and oxidative stress. Pharmacogn Mag. 2020; 16(Suppl 2):S236-245. doi: 10.4103/pm.pm_587_19

62. Yilmazer-Musa M, Griffith AM, Michels AJ, Schneider E, Frei B. Grape seed and tea extracts and catechin 3-gallates are potent inhibitors of α-amylase and α-glucosidase activity. J Agric Food Chem. 2012; 60(36):8924-8929. doi: 10.1021/jf301147n

63. Ghaffari S, Navabzadeh M, Ziaee M, Ghobadi A, Ghods R, Hashem-Dabaghian F. A randomized, triple-blind, placebo-controlled, add-on clinical trial to evaluate the efficacy of *Emblica officinalis* in uncontrolled hypertension. Evid Based Complementary Altern. Med. 2020; 2020:1-7. doi: 10.1155/2020/85928

Figure 1

Click here to access/download;Figure;Fig 1.tif

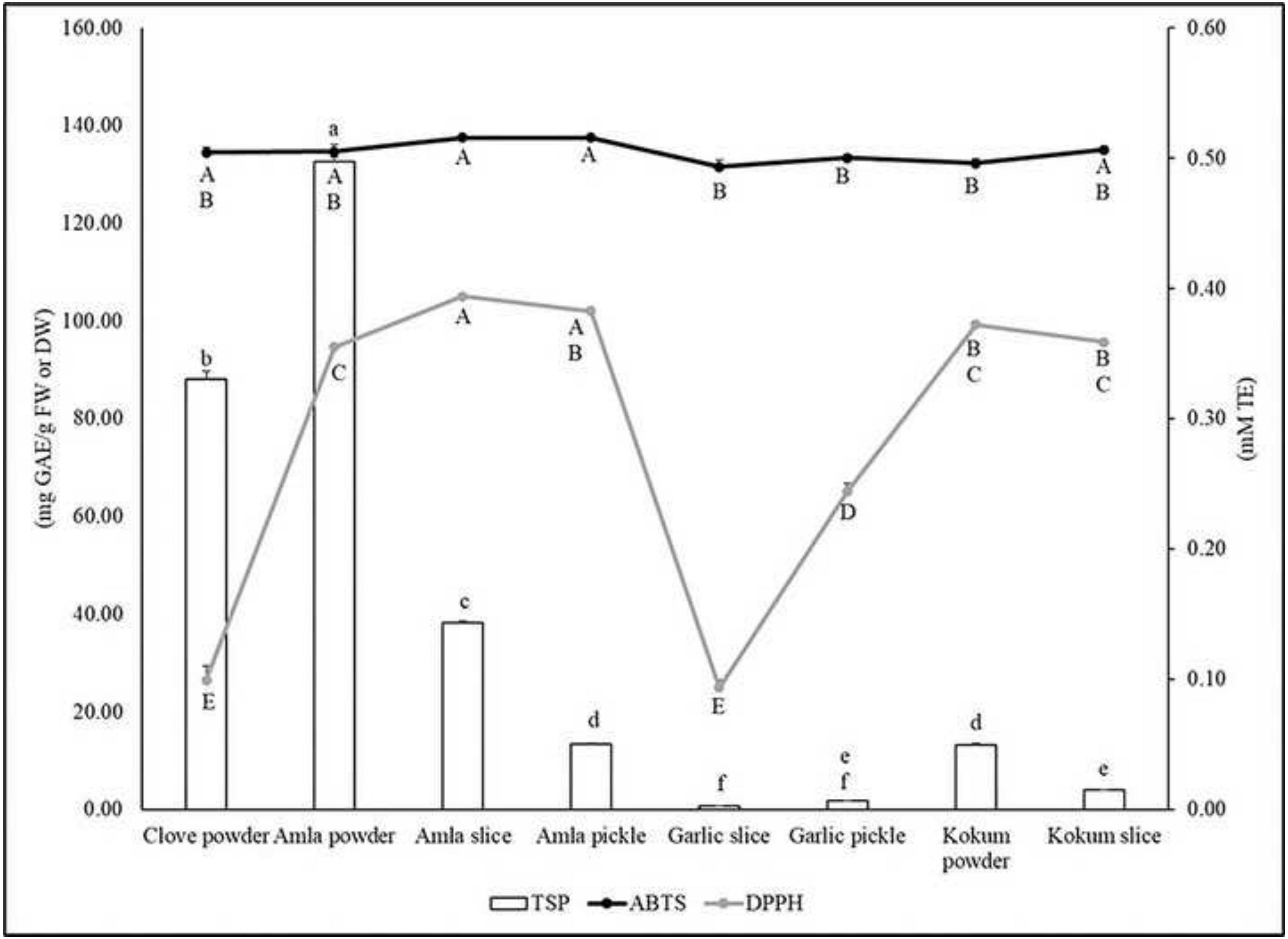

Figure 2

Click here to access/download;Figure;Fig 2.tif

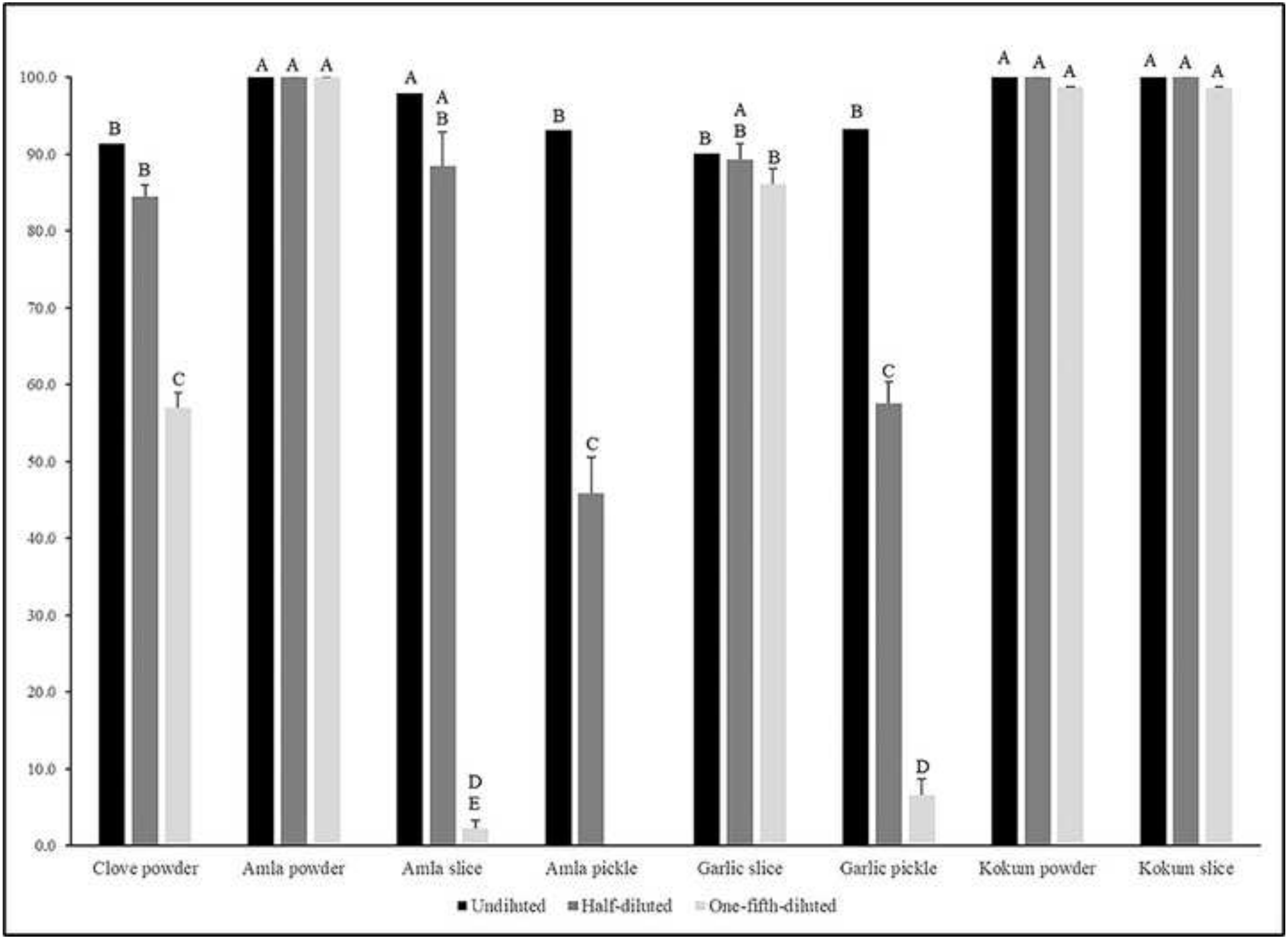

Supplement: Attachment — Submitted filename: PONE-D-24-12227_reviewer_KB 210424.pdf [file pone.0307807.s013.pdf]
